# Supplementary material for: Estimating the effectiveness of an enhanced ‘Improving Access to Psychological Therapies’ (IAPT) service addressing the wider determinants of mental health: a real-world evaluation
Source: BMJ Open. 2024 Jan 30;14(1):e077220. doi: 10.1136/bmjopen-2023-077220 (PMC10828880; doi:10.1136/bmjopen-2023-077220)
Supplement: Supplementary data [file bmjopen-2023-077220supp001.pdf]

Supplementary Figure 1. Diagram illustrating the enhanced Improving Access to Psychological Therapies service structure.

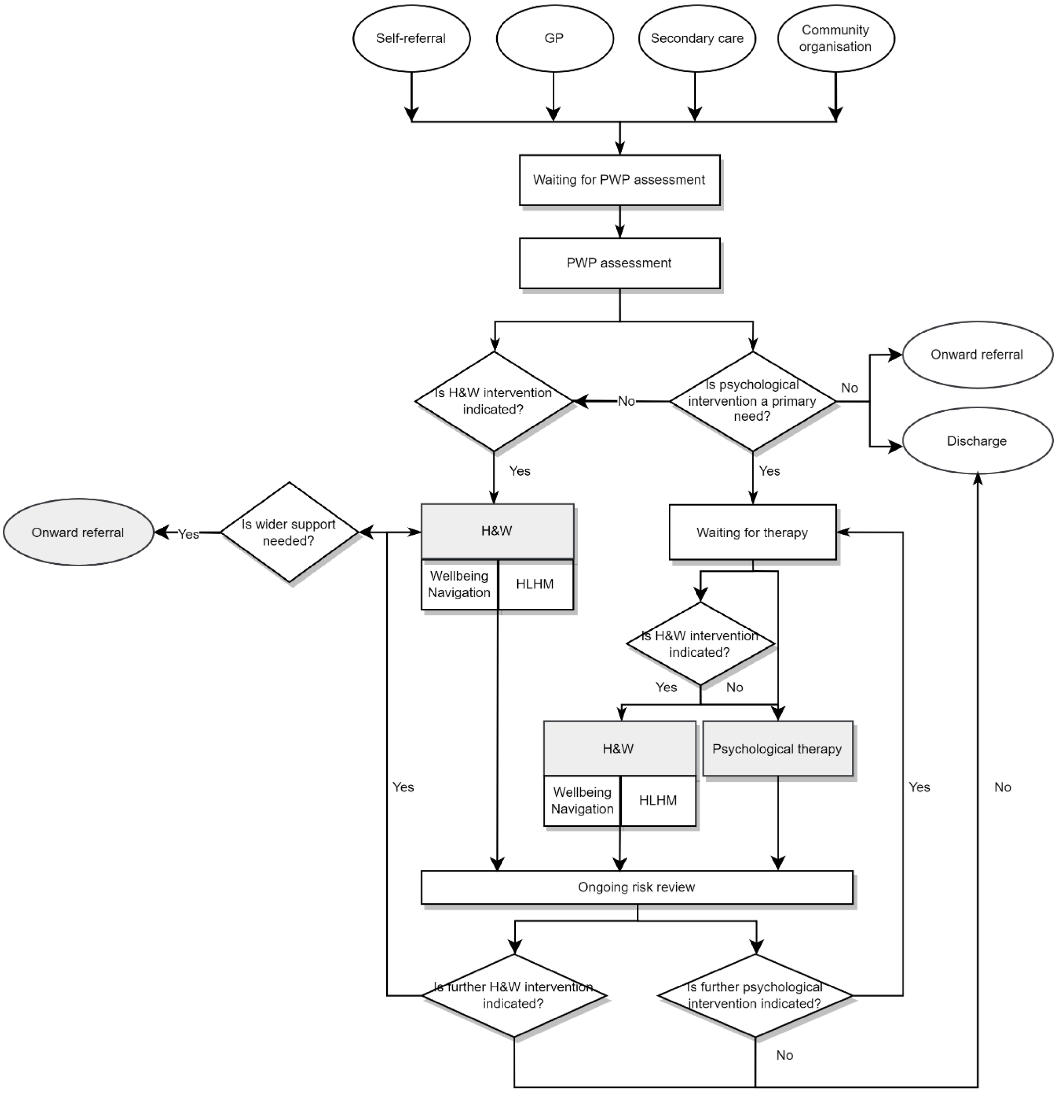

Grey shapes indicate the four types of support offered after PWP assessment; rectangle boxes indicate activities within IAPT; circles indicate activities outside of IAPT; diamonds indicate ongoing review questions.
Abbreviations: GP; General Practitioner, PWP; Psychological Wellbeing Practitioner, H&W; Health and Wellbeing Pathway, HLHM; Healthy Living Healthy Minds programme.

Supplementary Table 1. The RECORD statement for pharmacoepidemiology (RECORD-PE) checklist of items, extended from the STROBE and RECORD statements, which should be reported in non-interventional pharmacoepidemiologic studies using routinely collected health data

Item No	STROBE items	RECORD items	RECORD-PE items	Page No
**Title and abstract**				
1	(a) Indicate the study's design with a commonly used term in the title or the abstract.<br>(b) Provide in the abstract an informative and balanced summary of what was done and what was found.	1.1: The type of data used should be specified in the title or abstract. When possible, the name of the databases used should be included.<br>1.2: If applicable, the geographical region and timeframe within which the study took place should be reported in the title or abstract.<br>1.3: If linkage between databases was conducted for the study, this should be clearly stated in the title or abstract.	—	1-2
**Introduction**				
Background rationale				
2	Explain the scientific background and rationale for the investigation being reported.	—	—	4-5
Objectives				
3	State specific objectives, including any prespecified hypotheses.	—	—	5
**Methods**				
Study design				
4	Present key elements of study design early in the paper.	—	4.a: Include details of the specific study design (and its features) and report the use of multiple designs if used.<br>4.b: The use of a diagram(s) is recommended to illustrate key aspects of the study design(s), including exposure, washout, lag and observation periods, and covariate definitions as relevant.	5-6 Supplementary Figure 1
Setting				
5	Describe the setting, locations, and relevant dates, including periods of recruitment, exposure, follow-up, and data collection.	—	—	5-8
Participants				

6	(a) Cohort study—give the eligibility criteria, and the sources and methods of selection of participants. Describe methods of follow-up. Case-control study—give the eligibility criteria, and the sources and methods of case ascertainment and control selection. Give the rationale for the choice of cases and controls. Cross sectional study—give the eligibility criteria, and the sources and methods of selection of participants.<br>(b) Cohort study—for matched studies, give matching criteria and number of exposed and unexposed. Case-control study—for matched studies, give matching criteria and the number of controls per case.	6.1: The methods of study population selection (such as codes or algorithms used to identify participants) should be listed in detail. If this is not possible, an explanation should be provided.<br>6.2: Any validation studies of the codes or algorithms used to select the population should be referenced. If validation was conducted for this study and not published elsewhere, detailed methods and results should be provided.<br>6.3: If the study involved linkage of databases, consider use of a flow diagram or other graphical display to demonstrate the data linkage process, including the number of individuals with linked data at each stage.	6.1.a: Describe the study entry criteria and the order in which these criteria were applied to identify the study population. Specify whether only users with a specific indication were included and whether patients were allowed to enter the study population once or if multiple entries were permitted. See explanatory document for guidance related to matched designs.	8
Variables				
7	Clearly define all outcomes, exposures, predictors, potential confounders, and effect modifiers. Give diagnostic criteria, if applicable.	7.1: A complete list of codes and algorithms used to classify exposures, outcomes, confounders, and effect modifiers should be provided. If these cannot be reported, an explanation should be provided.	7.1.a: Describe how the drug exposure definition was developed.<br>7.1.b: Specify the data sources from which drug exposure information for individuals was obtained.<br>7.1.c: Describe the time window(s) during which an individual is considered exposed to the drug(s). The rationale for selecting a particular time window should be provided. The extent of potential left truncation or left censoring should be specified.<br>7.1.d: Justify how events are attributed to current, prior, ever, or cumulative drug exposure.<br>7.1.e: When examining drug dose and risk attribution, describe how current, historical or time on therapy are considered.<br>7.1.f: Use of any comparator groups should be outlined and justified.<br>7.1.g: Outline the approach used to handle individuals with more than one relevant drug exposure during the study period.	8-9
Data sources/measurement				
8	For each variable of interest, give sources of data and details of methods of assessment (measurement). Describe comparability of assessment methods if there is more than one group.	—	8.a: Describe the healthcare system and mechanisms for generating the drug exposure records. Specify the care setting in which the drug(s) of interest was prescribed.	8
Bias				

9	Describe any efforts to address potential sources of bias.	—	—	8<br>Table 1<br>Supplementary Figure 2
Study size				
10	Explain how the study size was arrived at.	—	—	8-9
Quantitative variables				
11	Explain how quantitative variables were handled in the analyses. If applicable, describe which groupings were chosen, and why.	—	—	9
Statistical methods				
12	(a) Describe all statistical methods, including those used to control for confounding.<br>(b) Describe any methods used to examine subgroups and interactions.<br>(c) Explain how missing data were addressed.<br>(d) Cohort study—if applicable, explain how loss to follow-up was addressed. Case-control study—if applicable, explain how matching of cases and controls was addressed. Cross sectional study—if applicable, describe analytical methods taking account of sampling strategy.<br>(e) Describe any sensitivity analyses.	—	12.1.a: Describe the methods used to evaluate whether the assumptions have been met.<br>12.1.b: Describe and justify the use of multiple designs, design features, or analytical approaches.	9-10
Data access and cleaning methods				
12	—	12.1: Authors should describe the extent to which the investigators had access to the database population used to create the study population.<br>12.2: Authors should provide information on the data cleaning methods used in the study.	—	9
Linkage				
12	—	12.3: State whether the study included person level, institutional level, or other data linkage across two or more databases. The methods of linkage and methods of linkage quality evaluation should be provided.	—	n/a
**Results**				
Participants				
13	(a) Report the numbers of individuals at each stage of the study (eg, numbers potentially eligible, examined for eligibility, confirmed eligible, included in the study, completing follow-up, and analysed).	13.1: Describe in detail the selection of the individuals included in the study (that is, study population selection) including filtering based on data quality, data availability, and	—	9<br>Supplementary Table 2

	(b) Give reasons for non-participation at each stage.<br>(c) Consider use of a flow diagram.	linkage. The selection of included individuals can be described in the text or by means of the study flow diagram.		
Descriptive data				
14	(a) Give characteristics of study participants (eg, demographic, clinical, social) and information on exposures and potential confounders.<br>(b) Indicate the number of participants with missing data for each variable of interest.<br>(c) Cohort study—summarise follow-up time (eg, average and total amount).	—	—	10<br>Table 2
Outcome data				
15	Cohort study—report numbers of outcome events or summary measures over time. Case-control study—report numbers in each exposure category, or summary measures of exposure. Cross sectional study—report numbers of outcome events or summary measures.	—	—	Table 2
Main results				
16	(a) Give unadjusted estimates and, if applicable, confounder adjusted estimates and their precision (eg, 95% confidence intervals). Make clear which confounders were adjusted for and why they were included.<br>(b) Report category boundaries when continuous variables are categorised.<br>(c) If relevant, consider translating estimates of relative risk into absolute risk for a meaningful time period.	—	—	12<br>Table 3
Other analyses				
17	Report other analyses done—eg, analyses of subgroups and interactions, and sensitivity analyses.	—	—	14<br>Table 4<br>Supplementary Table 3
**Discussion**				
Key results				
18	Summarise key results with reference to study objectives.	—	—	16-17
Limitations				
19	Discuss limitations of the study, taking into account sources of potential bias or imprecision. Discuss both direction and magnitude of any potential bias.	19.1: Discuss the implications of using data that were not created or collected to answer the specific research question(s). Include	19.1.a: Describe the degree to which the chosen database(s) adequately captures the drug exposure(s) of interest.	17-18

		discussion of misclassification bias, unmeasured confounding, missing data, and changing eligibility over time, as they pertain to the study being reported.		
Interpretation				
20	Give a cautious overall interpretation of results considering objectives, limitations, multiplicity of analyses, results from similar studies, and other relevant evidence.	—	20.a: Discuss the potential for confounding by indication, contraindication or disease severity or selection bias (healthy adherer/sick stopper) as alternative explanations for the study findings when relevant. **[A: Original text indicated this item was RECORD (ie, not RECORD-PE)?]**	19
Generalisability				
21	Discuss the generalisability (external validity) of the study results.	—	—	18
**Other information**				
Funding				
22	Give the source of funding and the role of the funders for the present study and, if applicable, for the original study on which the present article is based.	—	—	1
Accessibility of protocol, raw data, and programming code				
22	—	22.1: Authors should provide information on how to access any supplemental information such as the study protocol, raw data, or programming code.	—	n/a

Abbreviations: RECORD=reporting of studies conducted using observational routinely collected data; RECORD-PE=RECORD for pharmacoepidemiologic research; STROBE=strengthening the reporting of observational studies in epidemiology.

Supplementary Figure 2. Example box plots exploring the balance of covariates for age and baseline GAD-7 after propensity score estimation

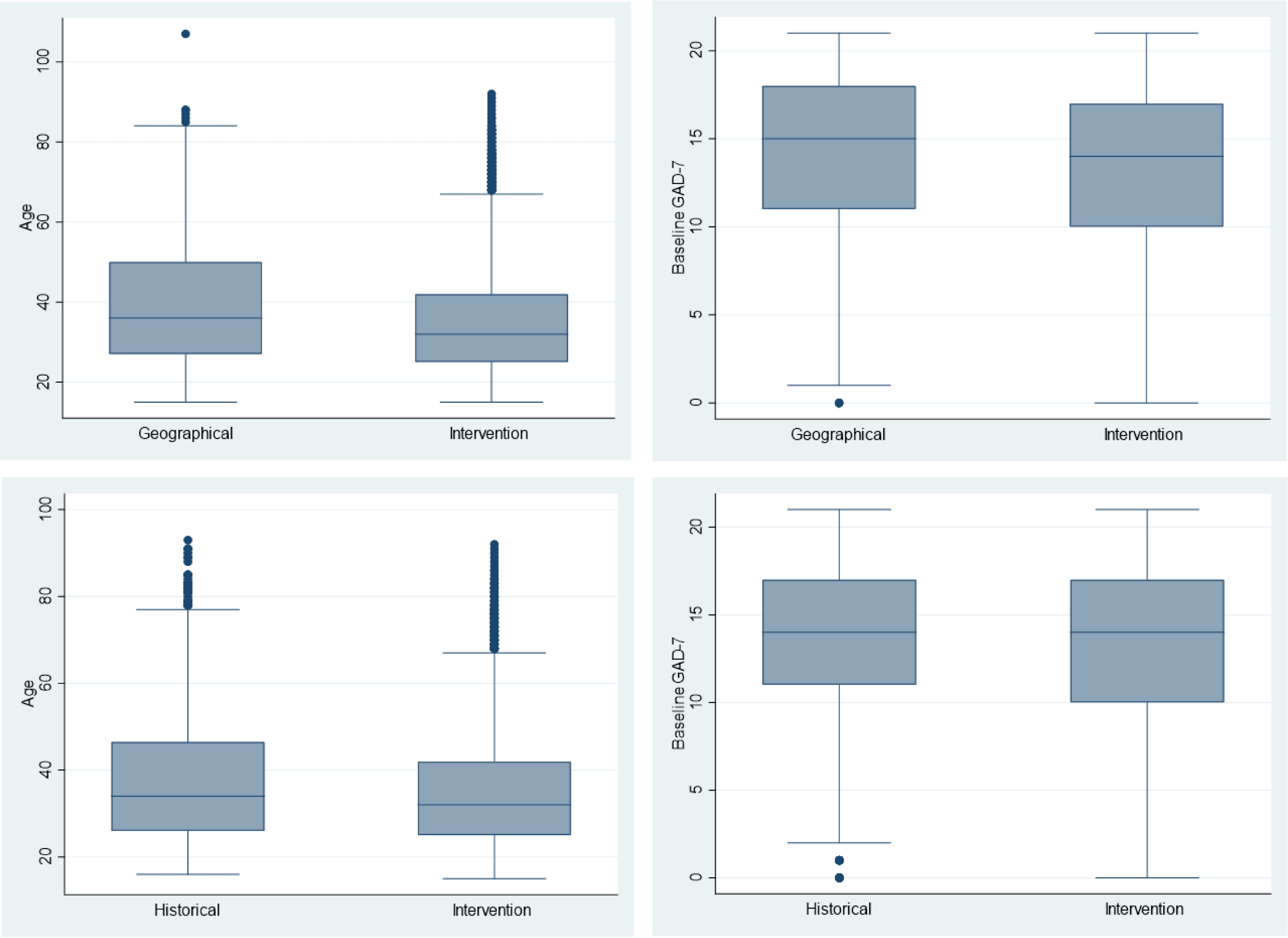

Supplementary Table 2. Number of service-users included in analyses

	Intervention group	Geographical-control group	Historical-control group
In IAPT EHR dataset	23,143	6865	12,045
Eligible for analysis	8020	3548	6074
Eligible for analysis and have been discharged from service (as-started analysis)	5853	2916	5372
Eligible for analysis, have been discharged from service and have been within service for at least 12 weeks	4284	1884	3698
Eligible for analysis and have been discharged from service and have been within service for at least 16 weeks	4529	1992	3738
Eligible for analysis and have been discharged from service and have been within service for at least 20 weeks	4153	1963	3630
Eligible for analysis and have been discharged from service and have been within service for at least 24 weeks	3747	1880	3287

Abbreviations: EHR; electronic health record

Supplementary Table 3. Doubly robust mixed effects linear regression models exploring group by demographic interactions effects for primary outcome in as-started sample

	Intervention vs Geographical-control n obs = 63,853, n patients = 11,271				Intervention vs Historical-control n obs = 79,685, n patients = 13,748			
	**Coefficient**	**95% CI**		**p value**	**Coefficient**	**95% CI**		**p value**
**PHQ-9**								
Group X Sex								
Intervention - Female	0.39	0.07	0.71	0.017	0.15	-0.11	0.42	0.256
Group X Age category								
Intervention - 29-40 years	0.09	-0.27	0.45	0.612	0.15	-0.14	0.45	0.310
Intervention - 41-91 years	0.00	-0.36	0.36	0.993	0.18	-0.13	0.49	0.261
Group X Ethnicity								
Intervention - Non-White	0.04	-0.48	0.56	0.881	-0.03	-0.45	0.39	0.881
Group X IMD category								
Intervention - Intermediate	0.24	-0.16	0.63	0.238	-0.20	-0.50	0.10	0.199
Intervention - High	0.14	-0.20	0.48	0.411	-0.24	-0.55	0.07	0.122
**GAD-7**								
Group X Sex								
Intervention - Female	0.38	0.09	0.66	0.010	0.11	-0.12	0.35	0.345
Group X Age category								
Intervention - 29-40 years	0.09	-0.24	0.42	0.595	0.08	-0.18	0.35	0.533
Intervention - 41-91 years	-0.01	-0.33	0.31	0.953	0.04	-0.23	0.32	0.763
Group X Ethnicity								
Intervention - Non-White	-0.04	-0.49	0.42	0.878	-0.13	-0.51	0.25	0.513
Group X IMD category								
Intervention - Intermediate	0.09	-0.26	0.44	0.610	-0.23	-0.50	0.04	0.089
Intervention - High	-0.08	-0.38	0.23	0.619	-0.30	-0.57	-0.02	0.034
**WSAS**								
Group X Sex								
Intervention - Female	0.39	-0.10	0.87	0.118	0.10	-0.30	0.49	0.634
Group X Age category								
Intervention - 29-40 years	-0.15	-0.71	0.40	0.586	-0.27	-0.72	0.17	0.222

Intervention - 41-91 years	-0.26	-0.79	0.26	0.325	0.06	-0.40	0.52	0.801
Group X Ethnicity								
Intervention - Non-White	-0.14	-0.97	0.69	0.737	0.08	-0.59	0.75	0.811
Group X IMD category								
Intervention - Intermediate	0.19	-0.40	0.78	0.533	-0.19	-0.64	0.26	0.414
Intervention - High	-0.03	-0.54	0.49	0.919	-0.53	-0.99	-0.07	0.023

All models include main effects for group, age, sex, ethnicity and IMD and are adjusted for baseline PHQ-9, baseline GAD-7 and baseline WSAS. Inverse propensity score weighting applied to all models, weighted for all covariates. Each group by demographic interaction model was run separately but are presented together in this table.

Sample includes all patients who met the study inclusion criteria, with follow up as the point of service discharge.
