# Supplementary material for: Estimating the effectiveness of an enhanced ‘Improving Access to Psychological Therapies’ (IAPT) service addressing the wider determinants of mental health: a real-world evaluation
Source: BMJ Open. 2024 Jan 30;14(1):e077220. doi: 10.1136/bmjopen-2023-077220 (PMC10828880; doi:10.1136/bmjopen-2023-077220)
Supplement: Author's manuscript [file bmjopen-2023-077220.draft_revisions.pdf]

BMJ Open is committed to open peer review. As part of this commitment we make the peer review history of every article we publish publicly available.

When an article is published we post the peer reviewers' comments and the authors' responses online. We also post the versions of the paper that were used during peer review. These are the versions that the peer review comments apply to.

The versions of the paper that follow are the versions that were submitted during the peer review process. They are not the versions of record or the final published versions. They should not be cited or distributed as the published version of this manuscript.

BMJ Open is an open access journal and the full, final, typeset and author-corrected version of record of the manuscript is available on our site with no access controls, subscription charges or pay-per-view fees (http://bmjopen.bmj.com).

If you have any questions on BMJ Open's open peer review process please email info.bmjopen@bmj.com

BMJ Open

# Estimating the effectiveness of an enhanced 'Improving Access to Psychological Therapies' (IAPT) service addressing the wider determinants of mental health: a real-world evaluation


| | |
|---|---|
| Journal: | *BMJ Open* |
| Manuscript ID | bmjopen-2023-077220 |
| Article Type: | Original research |
| Date Submitted by the Author: | 30-Jun-2023 |



| | |
|---|---|
| Complete List of Authors: | Porter, Alice; University of Bristol, Population Health Sciences<br>Franklin, Matthew; The University of Sheffield, Health Economic and Decision Science<br>De Vocht, Frank; University of Bristol, Population Health Sciences; NIHR Applied Research Collaboration West (NIHR ARC West)<br>d'Apice, Katrina; University of Bristol, Population Health Sciences<br>Curtin, Esther; University of Bristol, Population Health Sciences<br>Albers, Patricia ; University of Bristol, Population Health Sciences<br>Kidger, Judi; University of Bristol, Population Health Sciences |



| | |
|---|---|
| Keywords: | Adult psychiatry < PSYCHIATRY, MENTAL HEALTH, Electronic Health Records |
| | |


SCHOLARONE™
Manuscripts

*I, the Submitting Author has the right to grant and does grant on behalf of all authors of the Work (as defined in the below author licence), an exclusive licence and/or a non-exclusive licence for contributions from authors who are: i) UK Crown employees; ii) where BMJ has agreed a CC-BY licence shall apply, and/or iii) in accordance with the terms applicable for US Federal Government officers or employees acting as part of their official duties; on a worldwide, perpetual, irrevocable, royalty-free basis to BMJ Publishing Group Ltd ("BMJ") its licensees and where the relevant Journal is co-owned by BMJ to the co-owners of the Journal, to publish the Work in this journal and any other BMJ products and to exploit all rights, as set out in our licence.*

*The Submitting Author accepts and understands that any supply made under these terms is made by BMJ to the Submitting Author unless you are acting as an employee on behalf of your employer or a postgraduate student of an affiliated institution which is paying any applicable article publishing charge ("APC") for Open Access articles. Where the Submitting Author wishes to make the Work available on an Open Access basis (and intends to pay the relevant APC), the terms of reuse of such Open Access shall be governed by a Creative Commons licence – details of these licences and which Creative Commons licence will apply to this Work are set out in our licence referred to above.*

*Other than as permitted in any relevant BMJ Author's Self Archiving Policies, I confirm this Work has not been accepted for publication elsewhere, is not being considered for publication elsewhere and does not duplicate material already published. I confirm all authors consent to publication of this Work and authorise the granting of this licence.*

For peer review only

# Estimating the effectiveness of an enhanced 'Improving Access to Psychological Therapies' (IAPT) service addressing the wider determinants of mental health: a real-world evaluation

Alice Porter[1], Matthew Franklin[2], Frank de Vocht[1,3], Katrina d'Apice[1], Esther Curtin[4], Patricia N Albers[1], Judi Kidger[1]

[1] Centre for Public Health, Population Health Sciences, Bristol Medical School, University of Bristol, UK

[2] School for Health and Related Research (ScHARR), University of Sheffield, UK

[3] NIHR Applied Research Collaboration West (NIHR ARC West), Bristol, UK

[4] Department of Population Health, Faculty of Epidemiology and Population Health, London School of Hygiene and Tropical Medicine, UK

**Correspondence to:**

**Alice Porter,** Centre for Public Health, Population Health Sciences, Bristol Medical School, Canynge Hall, 39 Whatley Road, Bristol, BS8 2PS, UK

Word count: 3473

Contributors: JK, FDV, and MF were responsible for study conceptualisation and funding acquisition, PA, KD, FDV, MF, BK, AP and JK contributed to the analysis plan, AP conducted the analyses. AP wrote the first draft of the manuscript. All authors contributed to and approved the final manuscript.

Funding: This study is funded by the National Institute for Health and Care Research (NIHR) School for Public Health Research (SPHR), Grant Reference Number SPHR-PHPES004-IAPT. The views expressed are those of the author(s) and not necessarily those of the NIHR or the Department of Health and Social Care.



For peer review only - http://bmjopen.bmj.com/site/about/guidelines.xhtml

## ABSTRACT

**Background.** Addressing the wider determinants of mental health alongside psychological therapy could improve mental health service outcomes and population mental health.

**Objectives.** To estimate the effectiveness of an enhanced 'Improving Access to Psychological Therapies' (IAPT) mental health service compared to IAPT treatment-as-usual (TAU) in England. Alongside TAU, the enhanced service included wellbeing support and community service links.

**Methods.** A real-world evaluation using IAPT's electronic health records. We compared the enhanced IAPT service (intervention) to an IAPT service in a different region providing TAU only (geographical-control), and the IAPT service with TAU before additional support was introduced (historical-control). Primary outcomes were Patient Health Questionnaire-9 (PHQ-9) depression scale (score range: 0-27) and Generalised Anxiety Disorder-7 (GAD-7) anxiety scale (score range: 0-21); for both, lower scores indicate better mental health. Propensity scores were used to estimate inverse probability of treatment weights, subsequently used in mixed-effects regression models.

**Findings.** Small improvements (mean, 95% CI) were observed for PHQ-9 (depression) (-0.21, -0.32 to -0.09) and GAD-7 (anxiety) (-0.23, -0.34 to -0.13) scores in the intervention-group compared to the historical-control. There was little evidence of statistically significant differences between intervention and geographical-control.

**Discussion.** Embedding additional health and wellbeing support into standard IAPT services may lead to improved mental health outcomes. However, the lack of improved outcomes compared to the geographical-control may reflect a general improvement in all IAPT services.

**Clinical Implications.** It is not clear from our findings whether an IAPT service with additional health and wellbeing support is clinically superior to current standard IAPT models.


For peer review only



For peer review only - http://bmjopen.bmj.com/site/about/guidelines.xhtml

**What is already known on this topic**

- Empirical evidence suggests health and wellbeing support to address the wider determinants of mental health, when offered alongside psychological therapy, could help improve mental health outcomes.
- There is a lack of rigorous quantitative evaluations of mental health service redesigns based on real-world data such as electronic health records.

**What this study adds**

- This evaluation of an enhanced IAPT service in England provides limited evidence that offering health and wellbeing support leads to additional mental health benefits, over and above psychological therapy.

**How this study might affect research, practice or policy**

- Further research is required to establish how additional health and wellbeing support can be embedded into mental health services to improve service-user outcomes, compared to current standard services.
- Adaptations to local clinical mental health services should be continually evaluated to build an evidence-base about what changes can lead to additional benefits to service-users' mental health.



For peer review only - http://bmjopen.bmj.com/site/about/guidelines.xhtml

## INTRODUCTION

Approximately one in six adults in England have a diagnosable mental health disorder (MHD), for which prevalence and severity has worsened over the last 30 years, exacerbated by the COVID-19 pandemic.(1, 2) Preventing and treating MHDs effectively is a global public health priority.

Depression and anxiety are globally considered common MHDs, due to their prevalence.(3) Evidence-based effective treatment for common MHDs includes psychological therapies, e.g. cognitive behaviour therapy (CBT) or psychotherapy.(3) The National Health Service (NHS) England's *Improving Access to Psychological Therapies* (IAPT) programme provides evidence-based psychological therapies for MHDs. IAPT treatment starts with a GP referral or self-referral, with subsequent initial assessment by IAPT staff for anxiety and/or depression 'caseness', followed by allocation to the waiting-list before the first treatment session can be provided. Those requiring IAPT treatment are initially offered a multi-session course with a Psychological Wellbeing Practitioner (PWP), dependant on their symptom severity.(4)

IAPT defines anxiety and depression caseness as a service-user reported score of ≥8 on the Generalised Anxiety Disorder-7 questionnaire (GAD-7) and ≥10 on the Patient Health Questionnaire (PHQ-9), respectively. IAPT's patient-reported key performance indicators (KPIs) are based on the service-users' first (i.e. initial assessment) and last (i.e. at discharge) PHQ-9 and GAD-7 scores; this includes:

- *Recovery*: moving from 'caseness' (PHQ-9 ≥10; GAD-7 ≥8) on either measure to 'no caseness' (PHQ-9 <10; GAD-7 <8) on both measures.
- *Reliable change*: decrease of ≥6 on PHQ-9 or ≥4 on GAD-7, without score increase of PHQ-9 ≥6 or GAD-7 ≥4 on the other measure.
- *Reliable recovery*: achieving both recovery and reliable change.

Of 1.81 million adults referred to IAPT in 2021/22, approximately 50% were defined as 'recovered' at the point of service discharge.(5) However, a large proportion of people do not show reliable change or recover by the point of IAPT discharge. Additionally, common MHD prevalence has not reduced since the IAPT programme started in 2008.(6)

The World Health Organisation (WHO) suggests mental health services should consider psychosocial factors contributing to MHDs, e.g. stress, finances, difficulties at work, physical or mental abuse, and social support.(3) To address this, a 'whole person' approach to mental



For peer review only - http://bmjopen.bmj.com/site/about/guidelines.xhtml

health is required, including strengthening the links between IAPT and wider community and wellbeing support.(7, 8) A randomised controlled trial (RCT) found social prescribing (i.e., making referrals from a primary health care service to a voluntary community service) reduced anxiety and improved quality-of-life, but cost more and did not reduce depression severity.(9) An evaluation of IAPT linked with employment services reported that service-users who accessed employment support had better employment-related outcomes, with some indication of mental health improvements.(10)

As part of the mixed-methods 'Assessing a Distinct Improving Access to Psychological Therapies service' (ADAPT) study, we aimed to evaluate a new, locally enhanced IAPT service model, which introduced an additional health and wellbeing (H&W) pathway to address wider determinants of mental health. The aim of the quantitative evaluation reported in this article was to estimate and report the effectiveness of the enhanced IAPT service compared to IAPT Treatment-As-Usual (TAU).

## METHODS

### Intervention

The enhanced IAPT service (intervention) was introduced in March 2021 in three local authorities in South West (SW) England and replaced the existing IAPT service. After an initial assessment at baseline (i.e. before waiting-list allocation), service-users could be referred to IAPT TAU only (e.g., psychotherapy), IAPT TAU plus H&W pathway, or the H&W pathway alone. The H&W pathway consisted of two elements. First, the 'Healthy Living Healthy Minds' programme, a six-session group webinar series offering guided exercise and advice on healthy lifestyles. Secondly, and/or, one-to-one sessions with a 'Wellbeing Navigator', who facilitated access to community organisations to address the wider psychosocial problems individuals were experiencing (e.g., poverty, unemployment, and social isolation). Supplementary Figure 1 illustrates the enhanced IAPT service pathways.

### Study design and Target Trial protocol

A non-randomised study(11, 12) was conducted using IAPT's electronic health records (EHRs). Treatment allocation (at service-level) was non-randomised because it was not possible for the researchers to control its allocation, as the introduction of the enhanced IAPT service resulted from a new service provider and service redesign rather than a research study



For peer review only - http://bmjopen.bmj.com/site/about/guidelines.xhtml

design (e.g. RCT). The enhanced IAPT service (intervention) was compared to the previous IAPT service with TAU (historical-control), and to a standard IAPT service with TAU in South-East England (geographical-control). The historical-control was provided by a different service provider and the geographical-control by the same service provider as the intervention.

Table 1 provides an overview of the *a-priori* specified Target Trial (TT) framework protocol, which aims to reduce bias by clearly articulating seven study dimensions: eligibility criteria, treatment strategies, assignment procedure, follow-up period, outcomes, estimand(s), analysis plan.(12) The TT framework is intended to mimic the ideal 'pragmatic' RCT.

The Health Research Authority and Health and Care Research Wales granted ethical approval (reference number: 21/PR/0230). The study's planning, conducting and reporting follows the National Institute for Health and Care Excellence (NICE) Real-World Evidence (RWE) framework.(13) The RECORD-PE checklist is provided in the Supplementary Table 1.(14)



For peer review only - http://bmjopen.bmj.com/site/about/guidelines.xhtml

**Table 1. Target Trial protocol summary**

| Component | Description |
|---|---|
| **Eligibility criteria** | **New referrals to IAPT:** no attendance at the IAPT site in the previous 6-months since the new referral<br>**Newly referred during:** March 2021 to March 2022 (intervention & geographical control) or March 2018 to March 2019 (historical control)<br>**Condition caseness at baseline:** service-users classified as having depression caseness (PHQ-9≥10) or anxiety caseness (i.e. GAD-7≥8) at baseline (i.e. before waiting-list allocation)<br>**Baseline data:** recorded PHQ-9 (depression severity) and GAD-7 (anxiety severity) score at baseline – see ‘**Condition caseness at baseline**’<br>**As-started treatment:** service-users had attended at least one treatment session to be defined as ‘as-started’ treatment |
| **Treatment strategies** | **Intervention:** Enhanced IAPT service (South West, England), as treatment-as-usual (TAU) plus ‘Healthy Living Healthy Minds’ programme and/or 1:1 Wellbeing Navigator sessions.<br>**Geographical control:** TAU IAPT service in South East, England;<br>**Historical control:** TAU IAPT service in the intervention area but before the enhanced service had been implemented. |
| **Assignment procedures** | **Non-randomised and unblinded:** referrals are assessed for eligibility by the service, if deemed eligible are offered TAU (across all groups) or TAU plus ‘Healthy Living Healthy Minds’ programme and/or 1:1 Wellbeing Navigator sessions (intervention only), with uptake based on service-user preference. |
| **Follow-up period(s)** | Starts at baseline appointment to assess condition caseness and allocate people to the waiting-list before first treatment session, and ends at discharge from service or self-discharge from service. |
| **Outcome(s)** | **Primary:** PHQ-9 score (continuous), GAD-7 score (continuous), and WSAS score (continuous).<br>**Secondary:** reliable change (binary), recovery (binary variables), and waiting times (continuous variable). |
| **Estimand(s) (casual contrasts)** | **As-started primary:** in new referrals to IAPT, the between-group difference in mean PHQ-9 or GAD-7 or WSAS for those referred to the enhanced IAPT service compared to IAPT treatment-as-usual in the geographical or historical control at the point of service discharge, regardless of TAU received<br>**Sensitivity analyses:** in new referrals to IAPT, what is the between-group difference in mean PHQ-9 or GAD-7 or WSAS for those who have been within the service for at least 12 or 16 or 20 or 24 weeks, referred to the enhanced IAPT service compared to IAPT treatment-as-usual in the geographical or historical control at the point of service discharge, regardless of TAU received |
| **Analysis plan** | **Overall:** An analytical plan was pre-specified.<br>**Propensity scores:** logistic regression estimated propensity scores were used to derive inverse probability of treatment weights (IPTWs)<br>**Weighted regressions:** Linear mixed effects weighted-regression analyses were conducted for primary outcomes, with the weights based on IPTWs. Linear and logistic regression analyses were conducted for secondary outcomes. Unadjusted, fully adjusted and doubly robust (fully adjusted and weighted) model results were compared for each outcome.<br>**Sub-groups:** those within the intervention group who accessed: (1) the IAPT TAU only; (2) IAPT TAU plus the enhanced service (i.e., NB no service-users were offered the enhanced service without TAU).<br>**Health inequalities:** the primary outcome models were re-run with additional interactions between treatment group and age, gender, ethnicity, or IMD. |

Abbreviations: IAPT; Improving Access to Psychological Therapies, TAU; treatment-as-usual, PHQ-9; Patient Health Questionnaire-9, GAD-7; Generalized Anxiety Disorder questionnaire-7, WSAS; Work and Social Adjustment Scale



For peer review only

For peer review only - http://bmjopen.bmj.com/site/about/guidelines.xhtml

## Measures

We used IAPT's EHR data, anonymised before sharing (i.e. on IAPT premises) in line with information governance processes for secondary data analysis. Demographics (age, sex, ethnicity, postcode, and employment status) were provided as baseline variables. Postcodes were matched to a national Index of Multiple Deprivation (IMD) dataset, as an indicator of socioeconomic status (SES).(15) Primary outcomes included service-user reported scores as continuous variables:

- PHQ-9 depression score from 0 (best state) to 27 (worst state)
- GAD-7 anxiety scores from 0 (best state) to 21 (worst state)
- Work and Social Adjustment (WSAS) scale from 0 (best state) to 40 (worst state).

Secondary outcomes included reliable change (binary), recovery (binary) and waiting time (continuous). As per NHS England's IAPT manual,(16) *reliable change* and *recovery* (based on PHQ-9 and GAD-7 scores) were calculated and reported only for those who reached the point of service discharge. The 'waiting time' KPI was defined as the duration in days from referral to first appointment, as reported in the EHRs.

## Eligible participants

Eligible participants were referrals to IAPT classified as having depression (PHQ-9) and/or anxiety (GAD-7) 'caseness' at baseline, with 'baseline' defined as the initial assessment before waiting-list allocation, as part of TAU. For analyses focused on WSAS, eligible participants must have completed WSAS at baseline.

Intervention and geographical-controls were service-users in the relevant area who were referred between March 2021 and March 2022. Historical controls were service-users in the intervention area, referred between March 2018 and March 2019 (i.e. before the enhanced service was introduced when the service was just IAPT TAU); data from service-users referred between April 2019 and February 2021 were not requested, to minimise spill-over effects from the historical-control into the intervention group.

All eligible service-users had to have at least one treatment session to be defined as 'as-started' treatment: 'as-started' is the real-world study equivalent to a trial's intention-to-treat analysis, whereby the population group are defined as known to have started treatment regardless of subsequent treatment adherence (e.g., compared to per-protocol analyses, described as 'on-treatment' analyses for real-world studies).(13)



For peer review only - http://bmjopen.bmj.com/site/about/guidelines.xhtml

**Statistical analyses**

Descriptive and statistical analysis was conducted using Stata 15. Data preparation included recoding and deriving variables, and merging datasets required for analysis.

Propensity scores were estimated using logistic regression with treatment assignment as the dependent variable and clinical (baseline PHQ-9, GAD-7 and WSAS) and demographic variables (age, sex, IMD and ethnicity) as independent variables. The propensity scores were used to calculate inverse probability of treatment weights (IPTWs) used within mixed-effects regression models as a 'doubly robust' (i.e., weighted-regression) approach for balancing the comparison groups with respect to baseline covariates. This approach is defined as 'doubly robust' because combining the IPTWs and regression methods means only the weights or the regression need be correctly specified to obtain an unbiased effect estimator; thus minimising between-group baseline imbalances while accounting for baseline confounding due to the non-randomised treatment allocation.(17) Employment status was not included in the regression models due to the proportion of missing data (19%). Data were visually inspected to check the level of balance between groups after reweighting (see Supplementary Figure 2).

Linear mixed-effects weighted-regression analyses were conducted for the primary outcomes, to account for repeated measures, with service-user ID as the random effects variable. For the primary outcomes, a negative average treatment effect on the treated (ATT) represents an improvement to mental health. Linear and logistic weighted-regressions were conducted for secondary outcomes. Model residuals were inspected to test assumptions of distribution, linearity, and homoscedasticity. A complete-case analysis was conducted by focusing the analysis on people with at least one follow-up data collection time-point, thus assuming missing outcome scores were missing-completely-at-random. The follow-up period started at baseline and ended at discharge from the service.

**Sensitivity and sub-group analyses**

Sensitivity analyses were conducted to explore outcomes among samples of service users who had been within the service for at least 12, 16, 20 and 24-weeks, respectively, with the follow-up period ending at discharge from the service. These analyses were based on evidence suggesting at least 8-12 weeks of treatment is required to observe clinical change in mental health disorders.(18-20)



For peer review only - http://bmjopen.bmj.com/site/about/guidelines.xhtml

Sub-group analyses included those within the intervention group who accessed: (1) the IAPT TAU only; (2) IAPT TAU plus H&W pathway (NB: no service-users were offered the H&W pathway without TAU).

To explore potential health inequalities, the primary outcomes models were re-run with additional interactions between treatment-group and age, sex, ethnicity, or IMD. Effect sizes, 95% confidence intervals (CI) and p-values were reported.

## RESULTS

### Descriptive statistics

Table 2 presents demographic characteristics of the eligible participants, which were similar across intervention and control groups. Supplementary Table 2 presents the number of service-users with data available in the IAPT EHRs, who met study eligibility criteria, and who were included in analyses.

Table 2 also presents the mental health outcomes descriptively, showing that the mean baseline mental health scores were 1-2 points lower in the intervention vs control groups. The percentage of service-users achieving PHQ-9 reliable change were similar for intervention and historical-control (both 43%), but higher for geographical-controls (50%). Percentage of service-users achieving GAD-7 reliable change: intervention, 56%; geographical, 58%; historical, 52%. The percentage who recovered was similar for intervention and geographical-control (both 48%), but lower for historical-controls (38%).

For peer review only

**Table 2. Descriptive statistics for demographics and mental health measures scores for eligible sample**

| | | Intervention | | | | | | | | | Geographical | | | Historical | | |
|---|---|---|---|---|---|---|---|---|---|---|---|---|---|---|---|---|
| | | Intervention | | | IAPT TAU + H&W | | | IAPT TAU only | | | | | | | | |
| ***Measure*** | ***Sub-measure*** | ***N*** | ***%*** | | ***N*** | ***%*** | | ***N*** | ***%*** | | ***N*** | ***%*** | | ***N*** | ***%*** | |
| N overall | N/A | 8,020 | 100 | | 728 | 100 | | 7292 | 100 | | 3,548 | 100 | | 6,074 | 100 | |
| Sex | Female | 5,558 | 69 | | 494 | 68 | | 5,064 | 69 | | 2,461 | 69 | | 3,948 | 65 | |
| Ethnicity | Asian | 223 | 3 | | 30 | 4 | | 193 | 3 | | 105 | 3 | | 183 | 3 | |
| | Black | 175 | 2 | | 29 | 4 | | 146 | 2 | | 121 | 3 | | 175 | 3 | |
| | Mixed | 271 | 3 | | 26 | 4 | | 245 | 3 | | 88 | 2 | | 192 | 3 | |
| | Other | 100 | 1 | | 13 | 2 | | 87 | 1 | | 23 | 1 | | 62 | 1 | |
| | White | 7,109 | 89 | | 610 | 84 | | 6,499 | 89 | | 3,132 | 88 | | 5,336 | 88 | |
| Employment | Employed | 4,633 | 58 | | 382 | 52 | | 4,251 | 58 | | 1,812 | 51 | | 3,537 | 58 | |
| | Unemployed | 923 | 12 | | 163 | 22 | | 760 | 10 | | 758 | 21 | | 1,156 | 19 | |
| | Retired | 261 | 3 | | 37 | 5 | | 224 | 3 | | 235 | 7 | | 291 | 5 | |
| | Student | 679 | 8 | | 35 | 5 | | 644 | 9 | | 141 | 4 | | 397 | 7 | |
| PHQ-9 RC [a] | No | 3,346 | 57 | | 414 | 62 | | 2932 | 57 | | 1467 | 50 | | 3096 | 58 | |
| | Yes | 2,507 | 43 | | 256 | 38 | | 2251 | 43 | | 1449 | 50 | | 2276 | 42 | |
| GAD-7 RC [a] | No | 2,549 | 44 | | 350 | 52 | | 2199 | 42 | | 1226 | 42 | | 2594 | 48 | |
| | Yes | 3,304 | 56 | | 320 | 48 | | 2984 | 58 | | 1690 | 58 | | 2778 | 52 | |
| Recovered [a] | No | 3019 | 52 | | 485 | 72 | | 2534 | 49 | | 1507 | 52 | | 3342 | 62 | |
| | Yes | 2834 | 48 | | 185 | 28 | | 2649 | 51 | | 1409 | 48 | | 2030 | 38 | |
| ***Measure*** | ***Sub-measure*** | ***Mean*** | ***SD*** | ***Range*** | ***Mean*** | ***SD*** | ***Range*** | ***Mean*** | ***SD*** | ***Range*** | ***Mean*** | ***SD*** | ***Range*** | ***Mean*** | ***SD*** | ***Range*** |
| Age | N/A | 35 | 14 | 15-92 | 40 | 15 | 16-89 | 35 | 13 | 15-92 | 39 | 16 | 15-107 | 37 | 14 | 16-93 |
| IMD | Decile | 5.5 | 1.9 | - | 5.3 | 1.9 | - | 5.5 | 1.9 | - | 5.7 | 2.3 | - | 5.2 | 1.8 | 2.4-9.1 |
| PHQ-9 | Baseline | 15 | 5 | 0-27 | 17 | 5 | 1-27 | 14 | 5 | 0-27 | 16 | 5 | 0-27 | 16 | 5 | 0-27 |
| | Last obs. | 11 | 6 | 0-27 | 13 | 7 | 0-27 | 10 | 6 | 0-27 | 11 | 7 | 0-27 | 11 | 7 | 0-27 |
| | Dif. | -4.1 | 5.7 | -26 - 22 | -4.0 | 5.9 | -25 - 18 | -4.1 | 5.7 | -26 - 22 | -5.2 | 6.3 | -27 - 15 | -4.4 | 6 | -26 - 20 |
| GAD-7 | Baseline | 13 | 4 | 0-21 | 14 | 5 | 0-21 | 13 | 4 | 0-21 | 14 | 4 | 0-21 | 14 | 4 | 0-21 |
| | Last obs. | 9 | 6 | 0-21 | 11 | 6 | 0-21 | 9 | 6 | 0-21 | 10 | 6 | 0-21 | 10 | 6 | 0-21 |
| | Dif. | -3.9 | 5.4 | -21 - 17 | -3.4 | 5.3 | -21 - 14 | -4.0 | 5.4 | -21 - 17 | -4.7 | 5.8 | -21 - 14 | -3.9 | 5.7 | -21 - 19 |
| WSAS | Baseline | 18 | 8 | 0-40 | 21 | 9 | 0-40 | 18 | 8 | 0-40 | 19 | 9 | 0-40 | 20 | 9 | 0-40 |
| | Last obs. | 15 | 9 | 0-40 | 18 | 10 | 0-40 | 14 | 9 | 0-40 | 15 | 10 | 0-40 | 16 | 10 | 0-40 |
| | Dif. | -3.2 | 8.4 | -38 - 37 | -3.0 | 9.1 | -28 - 30 | -3.2 | 8.3 | -38 - 37 | -4.3 | 9.3 | -40 - 36 | -4.4 | 9.6 | -39 - 40 |
| Waiting time (days) | To 1st app. | 20 | 25 | 0-336 | 19 | 21 | 0-240 | 20 | 26 | 0-336 | 10 | 20 | 0 - 322 | 20 | 18 | 0-216 |
| No. sessions attended | To discharge | 5 | 3 | 1-32 | 8 | 6 | 1-32 | 5 | 3 | 1-27 | 5 | 3 | 1-25 | 6 | 4 | 1-30 |

Abbreviations: TAU; treatment-as-usual, H&W; Health & Wellbeing pathway, IMD; Index of Multiple Deprivation, SD; standard deviation PHQ-9; Physical Health Questionnaire-Depression Scale, GAD-7; Generalised Anxiety Disorder Questionnaire 7, WSAS; Work and Social Adjustment Scale, RC; Reliable change (defined as the reduction of six points or more on the PHQ-9 or four points or more on the GAD-7). Recovered, defined as moving below the threshold of ten on PHQ-9 or eight on GAD-7

[a] Includes patients who have been discharged from the service only.



For peer review only - http://bmjopen.bmj.com/site/about/guidelines.xhtml

**Primary analyses**

Table 3 indicates there was little difference in the ATT for PHQ-9, GAD-7 and WSAS when comparing the intervention to geographical-control. Compared to the historical-control, we observed a small, negative ATT for PHQ-9 (-0.40, 95% CI -0.53, -0.27), GAD-7 (-0.43, 95% CI -0.55, -0.32) and WSAS (-0.53, 95% CI -0.53, -0.15), representing small improvements in mental health in the intervention.

Table 3 indicates the odds of achieving PHQ-9 or GAD-7 reliable change were similar between intervention vs geographical-control. The odds of recovery were lower in the intervention vs geographical-control (OR=0.87, 95% CI 0.78, 0.96). Conversely, the odds were higher in the intervention vs historical-control for achieving PHQ-9 reliable change (OR=1.22, 95% CI 1.12, 1.33), GAD-7 reliable change (OR=1.17, 95% CI 1.17, 1.38), and recovery (OR=1.46, 95% CI 1.34, 1.59). Waiting-times were longer in the intervention vs geographical-control (mean = 10 days, 95% CI 9.21, 11.52) and marginally longer vs historical-control (mean = 1.5 days, 95% 0.67, 2.41).



For peer review only - http://bmjopen.bmj.com/site/about/guidelines.xhtml

245 **Table 3. Doubly robust mixed effects linear regression models for primary and secondary outcomes in as-started sample**

| | Intervention vs Geographical Control n obs = 48,652, n patients = 8,542 | | | | Intervention vs Historical Control n obs = 64,044, n patients = 10,951 | | | |
|---|---|---|---|---|---|---|---|---|
| | **Coefficient** | **95% CI** | | **p value** | **Coefficient** | **95% CI** | | **p value** |
| PHQ-9 | -0.08 | -0.23 | 0.07 | 0.284 | -0.40 | -0.53 | -0.27 | <0.001 |
| GAD-7 | -0.13 | -0.27 | 0.00 | 0.052 | -0.43 | -0.55 | -0.32 | <0.001 |
| WSAS | 0.09 | -0.14 | 0.32 | 0.437 | -0.34 | -0.53 | -0.15 | <0.001 |
| PHQ-9 reliable change | 0.90 | 0.82 | 1.00 | 0.045 | 1.22 | 1.12 | 1.33 | <0.001 |
| GAD-7 reliable change | 1.00 | 0.90 | 1.10 | 0.941 | 1.27 | 1.17 | 1.38 | <0.001 |
| Recovery | 0.87 | 0.78 | 0.96 | 0.007 | 1.46 | 1.34 | 1.59 | <0.001 |
| Waiting times | 10.37 | 9.21 | 11.52 | <0.001 | 1.54 | 0.67 | 2.41 | 0.001 |

246 All models are adjusted for covariates (age, gender, ethnicity, IMD, baseline PHQ-9, baseline GAD-7 and baseline WSAS). Inverse propensity score weighting applied to all
247 models, weighted for all covariates.
248 Sample includes all patients who met the study inclusion criteria, with follow up as the point of service discharge.
249 Reliable change PHQ-9 is defined as a reduction of ≥6 points. For patients to have a measure of reliable change PHQ-9 they must have been discharged.
250 Reliable change GAD-7 is defined as a reduction of ≥4 points. For patients to have a measure of reliable change GAD-7 they must have been discharged.
251 Recovery is defined as entering the service at the threshold of a score of ≥10 on PHQ-9 and/or ≥8 on GAD-7 and moving to "no-caseness", being a score below the threshold
252 on both measures of PHQ-9 and GAD-7. For a patient to have a measure of recovery they must have been discharged.



For peer review only - http://bmjopen.bmj.com/site/about/guidelines.xhtml

## Sensitivity analyses

Table 4 presents the sensitivity analysis results, which indicate no difference in ATT between intervention and geographical-control, except for a small negative ATT (i.e. improved score) for GAD-7 in the 24-week sample (-0.22, 95% CI -0.39, -0.05). Comparing the intervention to historical-control, small negative ATTs (i.e. improved scores) were observed for PHQ-9, GAD-7 and WSAS across all four samples, although associations were weak for WSAS in the 16-week to 24-week samples.

The odds of achieving PHQ-9 reliable change were higher in the intervention group vs geographical-control in the 24-week sample only (OR=1.15, 95% CI 1.02, 1.30). The odds of achieving GAD-7 reliable change were higher in the intervention group vs geographical control in the 20-week (OR=1.17, 95% CI 1.04, 1.32) and 24-week samples (OR=1.20, 95% CI 1.05, 1.36). The odds of recovery were not different between intervention and geographical-control. The odds of achieving PHQ-9 reliable change, GAD-7 reliable change and recovery were higher in the intervention vs historical-control across all four samples.

Across the four samples, findings suggest 10-12 days longer waiting-times in the intervention group compared to the geographical control group. Waiting times were slightly longer by 1.6-3.0 days in the intervention group compared to historical-control in the 16-week to 24-week samples, but no difference was observed in the 12-week sample.



For peer review only - http://bmjopen.bmj.com/site/about/guidelines.xhtml

**Table 4. Doubly robust mixed effects linear regression models for primary and secondary outcomes for patients within the service for at least 12-24 weeks**

| Measure | 12-weeks | | | | 16-weeks | | | | 20-weeks | | | | 24-weeks | | | |
|---|---|---|---|---|---|---|---|---|---|---|---|---|---|---|---|---|
| | Coef. | 95% CI | | p value | Coef. | 95% CI | | p value | Coef. | 95% CI | | p value | Coef. | 95% CI | | p value |
| | Intervention vs Geographical Control | | | | | | | | | | | | | | | |
| PHQ-9 | -0.11 | -0.29 | 0.07 | 0.229 | -0.13 | -0.31 | 0.04 | 0.131 | -0.10 | -0.28 | 0.08 | 0.268 | -0.19 | -0.38 | 0.01 | 0.056 |
| GAD-7 | -0.10 | -0.26 | 0.06 | 0.216 | -0.12 | -0.28 | 0.04 | 0.127 | -0.12 | -0.29 | 0.04 | 0.143 | -0.22 | -0.39 | -0.05 | 0.012 |
| WSAS | 0.20 | -0.07 | 0.46 | 0.141 | 0.03 | -0.24 | 0.29 | 0.833 | 0.00 | -0.27 | 0.28 | 0.978 | -0.10 | -0.39 | 0.18 | 0.476 |
| PHQ-9 RC | 1.05 | 0.93 | 1.18 | 0.459 | 1.06 | 0.94 | 1.19 | 0.351 | 1.12 | 0.99 | 1.26 | 0.062 | 1.15 | 1.02 | 1.30 | 0.027 |
| GAD-7 RC | 1.09 | 0.97 | 1.23 | 0.156 | 1.11 | 0.99 | 1.25 | 0.085 | 1.17 | 1.04 | 1.32 | 0.012 | 1.20 | 1.05 | 1.36 | 0.005 |
| Recovery | 1.07 | 0.94 | 1.21 | 0.288 | 1.03 | 0.91 | 1.16 | 0.670 | 1.13 | 1.00 | 1.28 | 0.053 | 1.11 | 0.98 | 1.26 | 0.113 |
| Waiting times | 10.04 | 9.03 | 11.05 | <0.001 | 10.88 | 9.94 | 11.81 | <0.001 | 11.23 | 10.36 | 12.10 | 0.000 | 11.06 | 10.19 | 11.94 | <0.001 |
| | Intervention vs Historical Control | | | | | | | | | | | | | | | |
| PHQ-9 | -0.46 | -0.61 | -0.31 | <0.001 | -0.35 | -0.50 | -0.20 | <0.001 | -0.32 | -0.48 | -0.17 | <0.001 | -0.31 | -0.48 | -0.15 | <0.001 |
| GAD-7 | -0.41 | -0.55 | -0.27 | <0.001 | -0.35 | -0.48 | -0.21 | <0.001 | -0.30 | -0.44 | -0.16 | <0.001 | -0.32 | -0.46 | -0.17 | <0.001 |
| WSAS | -0.39 | -0.61 | -0.17 | 0.001 | -0.27 | -0.49 | -0.05 | 0.019 | -0.25 | -0.48 | -0.02 | 0.036 | -0.25 | -0.50 | -0.01 | 0.043 |
| PHQ-9 RC | 1.26 | 1.14 | 1.40 | <0.001 | 1.16 | 1.05 | 1.28 | 0.003 | 1.13 | 1.02 | 1.24 | 0.018 | 1.11 | 1.00 | 1.23 | 0.045 |
| GAD-7 RC | 1.21 | 1.10 | 1.34 | <0.001 | 1.19 | 1.08 | 1.31 | <0.001 | 1.11 | 1.01 | 1.23 | 0.034 | 1.14 | 1.03 | 1.27 | 0.013 |
| Recovery | 1.47 | 1.33 | 1.64 | <0.001 | 1.34 | 1.22 | 1.49 | <0.001 | 1.34 | 1.21 | 1.49 | <0.001 | 1.30 | 1.17 | 1.45 | <0.001 |
| Waiting times | 0.30 | -0.59 | 1.19 | 0.511 | 1.57 | 0.75 | 2.39 | <0.001 | 2.49 | 1.71 | 3.27 | <0.001 | 3.01 | 2.22 | 3.79 | <0.001 |

Abbreviations: RC; reliable change

Intervention Vs. Geographical, N patient / N obs: 12-weeks, 6,021 / 35,999; 16-weeks, 6,364 / 37,965; 20-weeks, 5,972 / 36,310; 24-weeks, 5,499 / 33,829

Intervention Vs. Historical, N patient / N obs: 12-weeks, 7,805 / 47,512; 16-weeks, 8,076 / 50,071; 20-weeks, 7,603 / 47,835; 24-weeks, 6,880 / 43,873

All models are adjusted for covariates (age, gender, ethnicity, IMD, baseline PHQ-9, baseline GAD-7 and baseline WSAS). Inverse propensity score weighting applied to all models, weighted for all covariates.

Samples include all patients who met the study inclusion criteria and were in the service for at least 12, 16, 20, 24 weeks, respectively, with follow up as the point of service discharge.

Reliable change (RC) PHQ-9 is defined as a reduction of ≥6 points. For patients to have a measure of reliable change PHQ-9 they must have been discharged.

Reliable change (RC) GAD-7 is defined as a reduction of ≥4 points. For patients to have a measure of reliable change GAD-7 they must have been discharged.

Recovery is defined as entering the service at the threshold of a score of ≥10 on PHQ-9 and/or ≥8 on GAD-7 and moving to a "no-caseness", being a score below the threshold on both measures of PHQ-9 and GAD-7. For a patient to have a measure of recovery they must have been discharged.



For peer review only - http://bmjopen.bmj.com/site/about/guidelines.xhtml

### Sub-group analyses and investigating health inequalities

Table 2 shows that within the intervention group, the percentage of service-users achieving PHQ-9 or GAD-7 reliable change was lower among those who received IAPT TAU plus H&W pathway vs IAPT TAU only. However, Table 2 also shows that those who received IAPT TAU plus H&W pathway had slightly higher mean PHQ-9 and GAD-7 scores at baseline.

Supplementary Table 3 shows a group by gender interaction was observed for PHQ-9 and GAD-7 when comparing the intervention to geographical control, suggesting a slightly greater ATT among males in the geographical-control. A group by IMD interaction was observed for GAD-7 and WSAS when comparing intervention to historical-control, suggesting a slightly greater ATT among service users living in areas of low deprivation in the intervention group.

## DISCUSSION

Using a non-randomised study design and routinely collected 'real-world' IAPT EHR data, this study is the first to evaluate the effectiveness of providing an array of support to address the wider determinants of mental health in addition to psychological therapies. When comparing the intervention to the historical-control, our findings indicate that the enhanced service led to greater (albeit small) improvements in mental health scores and a higher number of service-users recovering from their mental disorder. However, when comparing the intervention to the geographical-control over the same time period, we observed little evidence of improvement.

Previous studies have shown IAPT service adaptation can address harmful health behaviours (e.g., smoking)(21) and help treat other mental health-related issues (e.g., insomnia).(22) This study provides evidence that IAPT services can be adapted to provide person-centred, tailored links to services, to address the underlying reasons for poor mental health, which meets key policy(7) and WHO recommendations.(3) However, in this study the evidence for an additional benefit over psychological therapy is limited, as we observed small improvements in the historical-control only. As the historical-control service was taken over by a new service provider - the same service provider as the geographical-control - it may be that the standard elements of the intervention service, such as the psychological therapy, had been improved by the new service provider, which could explain why we observe mental health improvements in the intervention compared to the historical-control but not the

For peer review only - http://bmjopen.bmj.com/site/about/guidelines.xhtml

geographical-control. In other words we cannot rule out that the effects seen were due to an improved standard service, rather than the enhanced aspects.

The reliable change and recovery results were more pronounced than the ATTs from the continuous outcome measure scores. The odds of achieving reliable change was between 11-27% and achieving recovery was between 30-47% greater in the intervention versus historical-control analyses, while the difference in mean PHQ-9 and GAD-7 scores between groups were minimal. Greater odds of achieving reliable change (15-20%) were also observed in the intervention versus geographical-control in the 24-week sensitivity analysis, but with no observed difference in mean PHQ-9 and GAD-7 scores. These findings suggest a large individual variability in treatment effectiveness and may partly explain the lack of average improvement observed at the group level. The reasons for this variability likely go beyond the data collected by IAPT (such as demographics and number of sessions attended), and may include factors such as service-user relationships with practitioners and linked services, and service-users' cognition, attitudes and beliefs.(23)

Previous evidence suggests 8-12 weeks is required to observe clinically meaningful changes for common MHDs.(18-20) However, our sensitivity analyses suggest treatment duration may have to be longer. We observed small but favourable intervention effects for anxiety severity and reliable change compared to the geographical-control among service-users receiving treatment for at least 24-weeks. A longer duration may be required to properly establish service-users' links into community services, and associated additional time from the point of addressing the wider determinants of people's mental health issues to the eventual impact on actual mental health outcome scores (e.g. PHQ-9 and GAD-7 scores).

Our findings suggest further adaptation or additional resources may be required to address potential health inequalities. Specifically, we found a greater improvement in mental health among service-users in the intervention compared to historical-controls, but only for those from a higher socioeconomic background. Relatedly, a systematic review and meta-analysis suggested socioeconomic deprivation was associated with poorer treatment outcomes for mental health.(24) Future research should explore and address the barriers faced by service-users living in deprived areas in achieving optimal treatment effects.

**Strengths and Limitations**

An RCT was not possible as the enhanced IAPT service was part of a local service redesign, thus treatment allocation could not be controlled by the researchers. Instead, we used a quasi-

For peer review only

experimental design based on real-world data (i.e., IAPTs EHRs) to estimate the effectiveness of the enhanced IAPT service. This approach has causal inference implications due to the observational nature of the study, which is more prone to bias (e.g., study-entry selection and confounding bias) than RCTs. However, real-world studies have benefits including representing the effectiveness of the intervention under real-world scenarios and using real-world data, which helps improve the external validity (e.g., generalisability) of the results within the setting of interest e.g. IAPT and NHS England. We used the TT framework (11) and NICE's RWE(13) framework to guide our study design and choice of analytical methods, including how we assessed and accounted for bias due to the non-randomised nature of the study. For example, we used a TT protocol, two control groups, and 'doubly robust' weighted-regression models, all of which are designed to reduce the potential impact of study-entry selection bias and confounding bias. Additionally, conducting pre-specified sensitivity analyses with secondary outcomes and different samples increased our ability to assess result robustness. However, as is common, we use a 'no unmeasured confounders' assumption, thus the potential impact of unmeasured confounding has not been fully assessed.(25)

Two control groups were used, however both have limitations to note: we cannot account for potential changes over time in the historical-control and for potential clustering effects in the geographical-control. Outcome score at service discharge was selected as the follow-up end-point to align with IAPT's KPIs of interest, i.e. based on PHQ-9 and GAD-7 scores at service discharge. This may have resulted in informative censoring because we do not know if and how service users' mental health may have changed after the point of discharge. In addition, service-users may have been discharged for reasons other than recovery, which was not indicated in IAPT's EHR. For example, early discharge could be due to mental health improvement, or self-discharge due to service dissatisfaction, both impacting on the service-users' outcome score at point of service discharge and beyond discharge. The use of more complex methods such as G-methods (e.g. inverse probability of censoring weighting) required to account for informative censoring were beyond the scope of this study, given these methods have commonly been applied more to time-to-event analyses related to survival than patient-reported outcome measure scores. The application of such methods to outcome measures is an area for future research.

In terms of data suitability, the IAPT's EHR data were preferable compared to primary data collection as it allowed for a large sample size, quicker evaluation, and minimal participant

For peer review only

burden. However, dropping variables and associated participants with too much missing data (e.g., employment status) reduced the study sample size and may have introduced some selection bias if our missing-completely-at-random assumption does not hold. In terms of intervention delivery, the enhanced service was newly introduced at the time of study, with the process of establishing links with wider community services still ongoing. This, in addition to the COVID-19 pandemic impacting service delivery, could have reduced the ability to link service-users to other support,(26) potentially reducing estimates of effectiveness.

**Clinical Implications**

There is evidence supporting the role of social prescribing through GPs(9) for individuals with physical and mental co-morbidities.(27) This study provides limited evidence that linking clinical mental health services, like IAPT, to public health community services addressing wider determinants of mental health may improve mental health outcomes. Stronger inferences cannot be made as we observed improvements in mental health when comparing the intervention to the historical-control but not to the geographical control. Longer term evaluation may be required to allow the enhanced IAPT service to be fully established for any impact to be clear.

**CONCLUSION**

This study provides evidence of an improved IAPT service over time. However, evidence is less clear that embedding additional health and wellbeing support leads to improved service-user mental health outcomes. Longer follow-up periods may be required to observe any further mental health improvements gained from health and wellbeing support in addition to psychological therapies.

For peer review only

Acknowledgements: We would like to thank the IAPT service providers for providing anonymised electronic health record data and information about the data used in our analyses.

Conflicts of interest: none

## REFERENCES

1. McManus S, Bebbington P, Jenkins R, et al. Mental health and wellbeing in England: Adult psychiatric morbidity survey 2014. 2016.
2. Daly M, Sutin AR, Robinson E. Longitudinal changes in mental health and the COVID-19 pandemic: evidence from the UK Household Longitudinal Study. Psychol Med. 2020:1-10.
3. World Health Organization. Mental Health 2019 [Available from: Mental health (who.int)]
4. NHS. The Improving Access to Psychological Therapies Manual. 2021. Contract No.: Gateway reference: 08101.
5. NHS Digital. Psychological Therapies, Annual report on the use of IAPT services, 2021-22. 2022.
6. Bastiampillai TJ, Allison S, Harford P, et al. Has the UK Improving Access to Psychological Therapies programme and rising antidepressant use had a public health impact? Lancet Psychiatry. 2019;6(3):e8-e9.
7. Griffiths S, Foster J, S. S, et al. Mental health's market experiment: Commissioning Psychological Therapies Through Any Qualified Provider. Chester: Centre for Psychological Therapies in Primary Care; 2013.
8. NHS England. The Community Mental Health Framework for Adults and Older Adults. 2019.
9. Grant C, Goodenough T, Harvey I, et al. A randomised controlled trial and economic evaluation of a referrals facilitator between primary care and the voluntary sector. BMJ. 2000;320(7232):419-23.
10. Hogarth T, Hasluck C, Gambin L, et al. Evaluation of Employment Advisors in the IAPT Programme. 2013.
11. de Vocht F, Katikireddi SV, McQuire C, et al. Conceptualising natural and quasi experiments in public health. BMC Med Res Methodol. 2021;21(1):32.
12. Hernan MA, Robins JM. Using Big Data to Emulate a Target Trial When a Randomized Trial Is Not Available. Am J Epidemiol. 2016;183(8):758-64.
13. NICE. NICE real-world evidence framework. 2022.
14. Langan SM, Schmidt SA, Wing K, et al. The reporting of studies conducted using observational routinely collected health data statement for pharmacoepidemiology (RECORD-PE). BMJ. 2018;363:k3532.
15. Ministry of Housing Communities & Local Government. English indices of deprivation 2019 2019 [Available from: https://imd-by-geo.opendatacommunities.org/imd/2019/area.
16. NHS England. The Improving Access to Psychological Therapies Manual. 2021.
17. Funk MJ, Westreich D, Wiesen C, et al. Doubly robust estimation of causal effects. Am J Epidemiol. 2011;173(7):761-7.
18. NICE. Depression in adults: recognition and management. London; 2009.
19. Ramsberg J, Asseburg C, Henriksson M. Effectiveness and cost-effectiveness of antidepressants in primary care: a multiple treatment comparison meta-analysis and cost-effectiveness model. PLoS One. 2012;7(8):e42003.

20. Annemans L, Brignone M, Druais S, et al. Cost-effectiveness analysis of pharmaceutical treatment options in the first-line management of major depressive disorder in Belgium. Pharmacoeconomics. 2014;32(5):479-93.
21. Taylor GMJ, Sawyer K, Kessler D, et al. Views about integrating smoking cessation treatment within psychological services for patients with common mental illness: A multi-perspective qualitative study. Health Expect. 2021;24(2):411-20.
22. Stott R, Pimm J, Emsley R, et al. Does adjunctive digital CBT for insomnia improve clinical outcomes in an improving access to psychological therapies service? Behav Res Ther. 2021;144:103922.
23. Martin LR, Williams SL, Haskard KB, et al. The challenge of patient adherence. Ther Clin Risk Manag. 2005;1(3):189-99.
24. Finegan M, Firth N, Wojnarowski C, et al. Associations between socioeconomic status and psychological therapy outcomes: A systematic review and meta-analysis. Depress Anxiety. 2018;35(6):560-73.
25. Austin PC. An Introduction to Propensity Score Methods for Reducing the Effects of Confounding in Observational Studies. Multivariate Behav Res. 2011;46(3):399-424.
26. Morris SL, Gibson K, Wildman JM, et al. Social prescribing during the COVID-19 pandemic: a qualitative study of service providers' and clients' experiences. BMC Health Serv Res. 2022;22(1):258.
27. Toffolutti V, Stuckler D, McKee M, et al. The employment and mental health impact of integrated Improving Access to Psychological Therapies: Evidence on secondary health care utilization from a pragmatic trial in three English counties. J Health Serv Res Policy. 2021;26(4):224-33.

For peer review only

**Supplementary Figure 1. Diagram illustrating the enhanced Improving Access to Psychological Therapies service structure.**

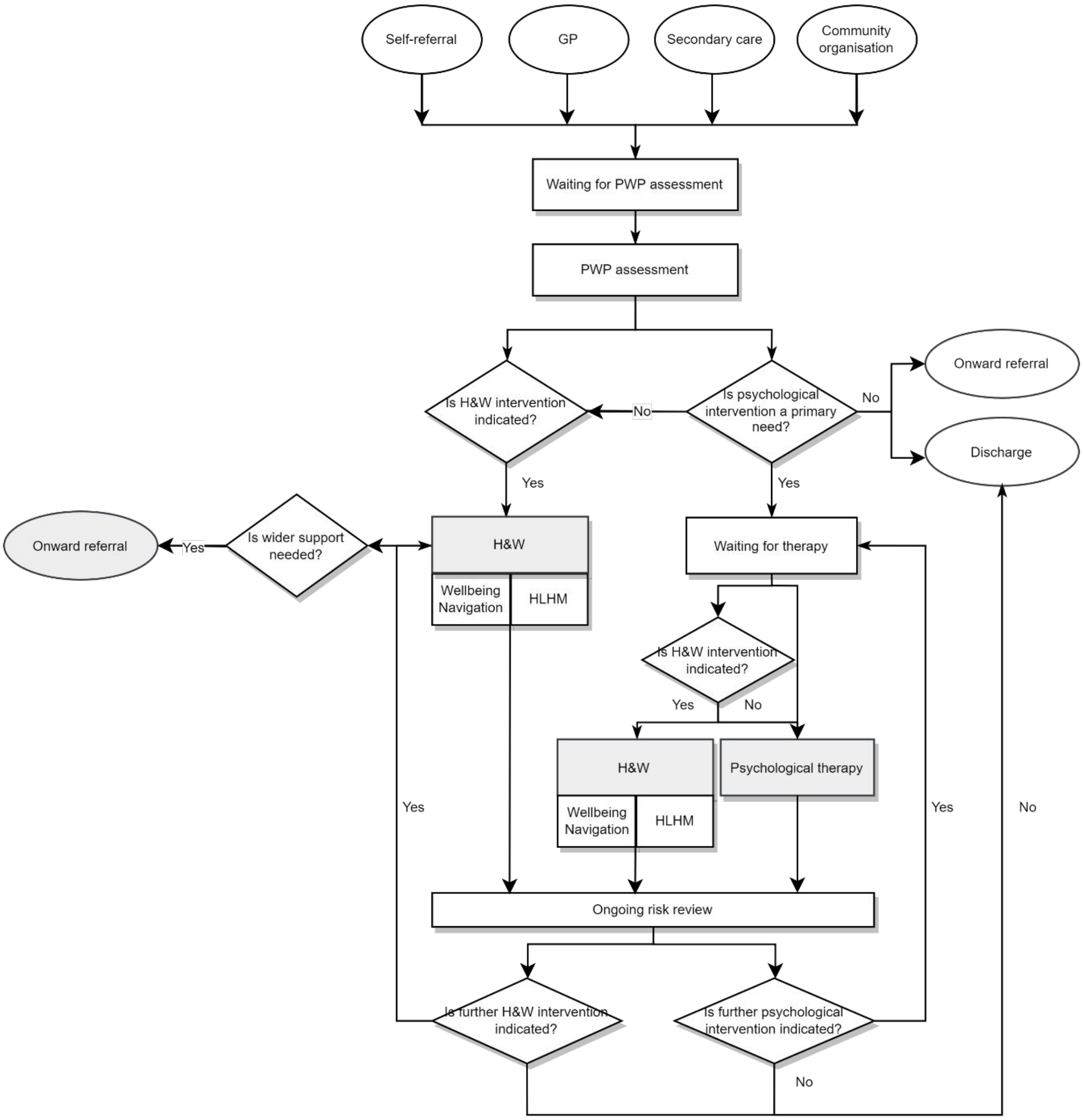


Grey shapes indicate the four types of support offered after PWP assessment; rectangle boxes indicate activities within IAPT; circles indicate activities outside of IAPT; diamonds indicate ongoing review questions.
Abbreviations: GP; General Practitioner, PWP; Psychological Wellbeing Practitioner, H&W; Health and Wellbeing Pathway, HLHM; Healthy Living Healthy Minds programme.

**Supplementary Table 1. The RECORD statement for pharmacoepidemiology (RECORD-PE) checklist of items, extended from the STROBE and RECORD statements, which should be reported in non-interventional pharmacoepidemiologic studies using routinely collected health data**

| Item No | STROBE items | RECORD items | RECORD-PE items | Page No |
|---|---|---|---|---|
| **Title and abstract** | | | | |
| 1 | (a) Indicate the study's design with a commonly used term in the title or the abstract.<br>(b) Provide in the abstract an informative and balanced summary of what was done and what was found. | 1.1: The type of data used should be specified in the title or abstract. When possible, the name of the databases used should be included.<br>1.2: If applicable, the geographical region and timeframe within which the study took place should be reported in the title or abstract.<br>1.3: If linkage between databases was conducted for the study, this should be clearly stated in the title or abstract. | — | 1-2 |
| **Introduction** | | | | |
| Background rationale | | | | |
| 2 | Explain the scientific background and rationale for the investigation being reported. | — | — | 4-5 |
| Objectives | | | | |
| 3 | State specific objectives, including any prespecified hypotheses. | — | — | 5 |
| **Methods** | | | | |
| Study design | | | | |
| 4 | Present key elements of study design early in the paper. | — | 4.a: Include details of the specific study design (and its features) and report the use of multiple designs if used.<br>4.b: The use of a diagram(s) is recommended to illustrate key aspects of the study design(s), including exposure, washout, lag and observation periods, and covariate definitions as relevant. | 5-6 Supplementary Figure 1 |
| Setting | | | | |
| 5 | Describe the setting, locations, and relevant dates, including periods of recruitment, exposure, follow-up, and data collection. | — | — | 5-8 |
| Participants | | | | |

For peer review only

| | | | | |
|---|---|---|---|---|
| 6 | (a) Cohort study—give the eligibility criteria, and the sources and methods of selection of participants. Describe methods of follow-up. Case-control study—give the eligibility criteria, and the sources and methods of case ascertainment and control selection. Give the rationale for the choice of cases and controls. Cross sectional study—give the eligibility criteria, and the sources and methods of selection of participants.<br>(b) Cohort study—for matched studies, give matching criteria and number of exposed and unexposed. Case-control study—for matched studies, give matching criteria and the number of controls per case. | 6.1: The methods of study population selection (such as codes or algorithms used to identify participants) should be listed in detail. If this is not possible, an explanation should be provided.<br>6.2: Any validation studies of the codes or algorithms used to select the population should be referenced. If validation was conducted for this study and not published elsewhere, detailed methods and results should be provided.<br>6.3: If the study involved linkage of databases, consider use of a flow diagram or other graphical display to demonstrate the data linkage process, including the number of individuals with linked data at each stage. | 6.1.a: Describe the study entry criteria and the order in which these criteria were applied to identify the study population. Specify whether only users with a specific indication were included and whether patients were allowed to enter the study population once or if multiple entries were permitted. See explanatory document for guidance related to matched designs. | 8 |
| Variables | | | | |
| 7 | Clearly define all outcomes, exposures, predictors, potential confounders, and effect modifiers. Give diagnostic criteria, if applicable. | 7.1: A complete list of codes and algorithms used to classify exposures, outcomes, confounders, and effect modifiers should be provided. If these cannot be reported, an explanation should be provided. | 7.1.a: Describe how the drug exposure definition was developed.<br>7.1.b: Specify the data sources from which drug exposure information for individuals was obtained.<br>7.1.c: Describe the time window(s) during which an individual is considered exposed to the drug(s). The rationale for selecting a particular time window should be provided. The extent of potential left truncation or left censoring should be specified.<br>7.1.d: Justify how events are attributed to current, prior, ever, or cumulative drug exposure.<br>7.1.e: When examining drug dose and risk attribution, describe how current, historical or time on therapy are considered.<br>7.1.f: Use of any comparator groups should be outlined and justified.<br>7.1.g: Outline the approach used to handle individuals with more than one relevant drug exposure during the study period. | 8-9 |
| Data sources/measurement | | | | |
| 8 | For each variable of interest, give sources of data and details of methods of assessment (measurement). Describe comparability of assessment methods if there is more than one group. | — | 8.a: Describe the healthcare system and mechanisms for generating the drug exposure records. Specify the care setting in which the drug(s) of interest was prescribed. | 8 |
| Bias | | | | |

| | | | | |
|---|---|---|---|---|
| 9 | Describe any efforts to address potential sources of bias. | — | — | 8<br>Table 1<br>Supplementary Figure 2 |
| Study size | | | | |
| 10 | Explain how the study size was arrived at. | — | — | 8-9 |
| Quantitative variables | | | | |
| 11 | Explain how quantitative variables were handled in the analyses. If applicable, describe which groupings were chosen, and why. | — | — | 9 |
| Statistical methods | | | | |
| 12 | (a) Describe all statistical methods, including those used to control for confounding.<br>(b) Describe any methods used to examine subgroups and interactions.<br>(c) Explain how missing data were addressed.<br>(d) Cohort study—if applicable, explain how loss to follow-up was addressed. Case-control study—if applicable, explain how matching of cases and controls was addressed. Cross sectional study—if applicable, describe analytical methods taking account of sampling strategy.<br>(e) Describe any sensitivity analyses. | — | 12.1.a: Describe the methods used to evaluate whether the assumptions have been met.<br>12.1.b: Describe and justify the use of multiple designs, design features, or analytical approaches. | 9-10 |
| Data access and cleaning methods | | | | |
| 12 | — | 12.1: Authors should describe the extent to which the investigators had access to the database population used to create the study population.<br>12.2: Authors should provide information on the data cleaning methods used in the study. | — | 9 |
| Linkage | | | | |
| 12 | — | 12.3: State whether the study included person level, institutional level, or other data linkage across two or more databases. The methods of linkage and methods of linkage quality evaluation should be provided. | — | n/a |
| **Results** | | | | |
| Participants | | | | |
| 13 | (a) Report the numbers of individuals at each stage of the study (eg, numbers potentially eligible, examined for eligibility, confirmed eligible, included in the study, completing follow-up, and analysed). | 13.1: Describe in detail the selection of the individuals included in the study (that is, study population selection) including filtering based on data quality, data availability, and | — | 9<br>Supplementary Table 2 |

For peer review only

| | | | | |
|---|---|---|---|---|
| | (b) Give reasons for non-participation at each stage.<br>(c) Consider use of a flow diagram. | linkage. The selection of included individuals can be described in the text or by means of the study flow diagram. | | |
| Descriptive data | | | | |
| 14 | (a) Give characteristics of study participants (eg, demographic, clinical, social) and information on exposures and potential confounders.<br>(b) Indicate the number of participants with missing data for each variable of interest.<br>(c) Cohort study—summarise follow-up time (eg, average and total amount). | — | — | 10<br>Table 2 |
| Outcome data | | | | |
| 15 | Cohort study—report numbers of outcome events or summary measures over time. Case-control study—report numbers in each exposure category, or summary measures of exposure. Cross sectional study—report numbers of outcome events or summary measures. | — | — | Table 2 |
| Main results | | | | |
| 16 | (a) Give unadjusted estimates and, if applicable, confounder adjusted estimates and their precision (eg, 95% confidence intervals). Make clear which confounders were adjusted for and why they were included.<br>(b) Report category boundaries when continuous variables are categorised.<br>(c) If relevant, consider translating estimates of relative risk into absolute risk for a meaningful time period. | — | — | 12<br>Table 3 |
| Other analyses | | | | |
| 17 | Report other analyses done—eg, analyses of subgroups and interactions, and sensitivity analyses. | — | — | 14<br>Table 4<br>Supplementary Table 3 |
| **Discussion** | | | | |
| Key results | | | | |
| 18 | Summarise key results with reference to study objectives. | — | — | 16-17 |
| Limitations | | | | |
| 19 | Discuss limitations of the study, taking into account sources of potential bias or imprecision. Discuss both direction and magnitude of any potential bias. | 19.1: Discuss the implications of using data that were not created or collected to answer the specific research question(s). Include | 19.1.a: Describe the degree to which the chosen database(s) adequately captures the drug exposure(s) of interest. | 17-18 |

For peer review only

| | | | | |
|---|---|---|---|---|
| | | discussion of misclassification bias, unmeasured confounding, missing data, and changing eligibility over time, as they pertain to the study being reported. | | |
| Interpretation | | | | |
| 20 | Give a cautious overall interpretation of results considering objectives, limitations, multiplicity of analyses, results from similar studies, and other relevant evidence. | — | 20.a: Discuss the potential for confounding by indication, contraindication or disease severity or selection bias (healthy adherer/sick stopper) as alternative explanations for the study findings when relevant. **[A: Original text indicated this item was RECORD (ie, not RECORD-PE)?]** | 19 |
| Generalisability | | | | |
| 21 | Discuss the generalisability (external validity) of the study results. | — | — | 18 |
| **Other information** | | | | |
| Funding | | | | |
| 22 | Give the source of funding and the role of the funders for the present study and, if applicable, for the original study on which the present article is based. | — | — | 1 |
| Accessibility of protocol, raw data, and programming code | | | | |
| 22 | — | 22.1: Authors should provide information on how to access any supplemental information such as the study protocol, raw data, or programming code. | — | n/a |

Abbreviations: RECORD=reporting of studies conducted using observational routinely collected data; RECORD-PE=RECORD for pharmacoepidemiologic research; STROBE=strengthening the reporting of observational studies in epidemiology.

For peer review only

**Supplementary Figure 2. Example box plots exploring the balance of covariates for age and baseline GAD-7 after propensity score estimation**

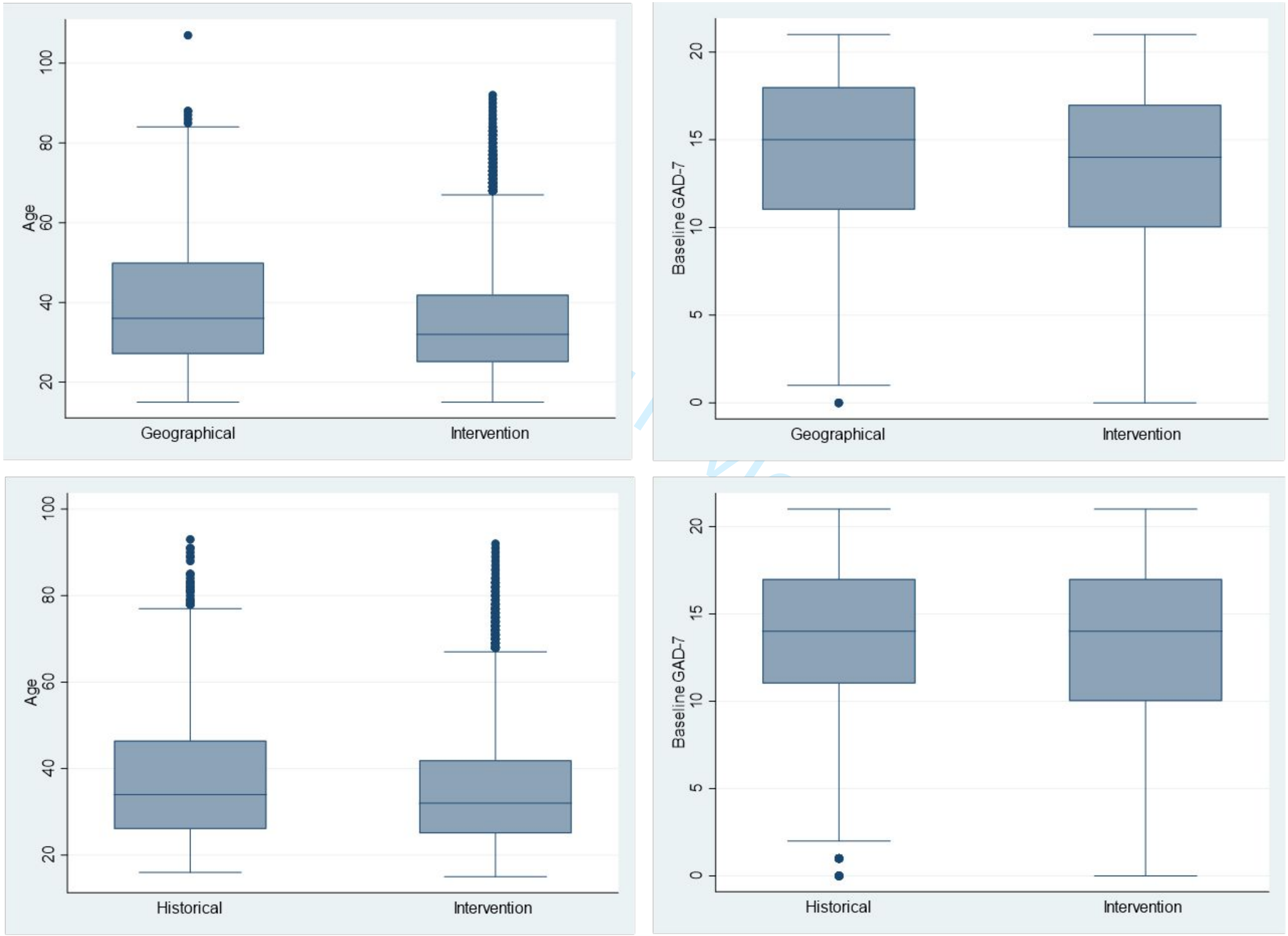

**Supplementary Table 2. Number of service-users included in analyses**

| | Intervention group | Geographical-control group | Historical-control group |
|---|---|---|---|
| In IAPT EHR dataset | 23,143 | 6865 | 12,045 |
| Eligible for analysis | 8020 | 3548 | 6074 |
| Eligible for analysis and have been discharged from service (as-started analysis) | 5853 | 2916 | 5372 |
| Eligible for analysis, have been discharged from service and have been within service for at least 12 weeks | 4284 | 1884 | 3698 |
| Eligible for analysis and have been discharged from service and have been within service for at least 16 weeks | 4529 | 1992 | 3738 |
| Eligible for analysis and have been discharged from service and have been within service for at least 20 weeks | 4153 | 1963 | 3630 |
| Eligible for analysis and have been discharged from service and have been within service for at least 24 weeks | 3747 | 1880 | 3287 |

Abbreviations: EHR; electronic health record

For peer review only

**Supplementary Table 3. Doubly robust mixed effects linear regression models exploring group by demographic interactions effects for primary outcome in as-started sample**

| | Intervention vs Geographical-control n obs = 63,853, n patients = 11,271 | | | | Intervention vs Historical-control n obs = 79,685, n patients = 13,748 | | | |
|---|---|---|---|---|---|---|---|---|
| | **Coefficient** | **95% CI** | | **p value** | **Coefficient** | **95% CI** | | **p value** |
| **PHQ-9** | | | | | | | | |
| Group X Sex | | | | | | | | |
| Intervention - Female | 0.39 | 0.07 | 0.71 | 0.017 | 0.15 | -0.11 | 0.42 | 0.256 |
| Group X Age category | | | | | | | | |
| Intervention - 29-40 years | 0.09 | -0.27 | 0.45 | 0.612 | 0.15 | -0.14 | 0.45 | 0.310 |
| Intervention - 41-91 years | 0.00 | -0.36 | 0.36 | 0.993 | 0.18 | -0.13 | 0.49 | 0.261 |
| Group X Ethnicity | | | | | | | | |
| Intervention - Non-White | 0.04 | -0.48 | 0.56 | 0.881 | -0.03 | -0.45 | 0.39 | 0.881 |
| Group X IMD category | | | | | | | | |
| Intervention - Intermediate | 0.24 | -0.16 | 0.63 | 0.238 | -0.20 | -0.50 | 0.10 | 0.199 |
| Intervention - High | 0.14 | -0.20 | 0.48 | 0.411 | -0.24 | -0.55 | 0.07 | 0.122 |
| **GAD-7** | | | | | | | | |
| Group X Sex | | | | | | | | |
| Intervention - Female | 0.38 | 0.09 | 0.66 | 0.010 | 0.11 | -0.12 | 0.35 | 0.345 |
| Group X Age category | | | | | | | | |
| Intervention - 29-40 years | 0.09 | -0.24 | 0.42 | 0.595 | 0.08 | -0.18 | 0.35 | 0.533 |
| Intervention - 41-91 years | -0.01 | -0.33 | 0.31 | 0.953 | 0.04 | -0.23 | 0.32 | 0.763 |
| Group X Ethnicity | | | | | | | | |
| Intervention - Non-White | -0.04 | -0.49 | 0.42 | 0.878 | -0.13 | -0.51 | 0.25 | 0.513 |
| Group X IMD category | | | | | | | | |
| Intervention - Intermediate | 0.09 | -0.26 | 0.44 | 0.610 | -0.23 | -0.50 | 0.04 | 0.089 |
| Intervention - High | -0.08 | -0.38 | 0.23 | 0.619 | -0.30 | -0.57 | -0.02 | 0.034 |
| **WSAS** | | | | | | | | |
| Group X Sex | | | | | | | | |
| Intervention - Female | 0.39 | -0.10 | 0.87 | 0.118 | 0.10 | -0.30 | 0.49 | 0.634 |
| Group X Age category | | | | | | | | |
| Intervention - 29-40 years | -0.15 | -0.71 | 0.40 | 0.586 | -0.27 | -0.72 | 0.17 | 0.222 |

For peer review only

| | | | | | | | | |
|---|---|---|---|---|---|---|---|---|
| Intervention - 41-91 years | -0.26 | -0.79 | 0.26 | 0.325 | 0.06 | -0.40 | 0.52 | 0.801 |
| Group X Ethnicity | | | | | | | | |
| Intervention - Non-White | -0.14 | -0.97 | 0.69 | 0.737 | 0.08 | -0.59 | 0.75 | 0.811 |
| Group X IMD category | | | | | | | | |
| Intervention - Intermediate | 0.19 | -0.40 | 0.78 | 0.533 | -0.19 | -0.64 | 0.26 | 0.414 |
| Intervention - High | -0.03 | -0.54 | 0.49 | 0.919 | -0.53 | -0.99 | -0.07 | 0.023 |

All models include main effects for group, age, sex, ethnicity and IMD and are adjusted for baseline PHQ-9, baseline GAD-7 and baseline WSAS. Inverse propensity score weighting applied to all models, weighted for all covariates. Each group by demographic interaction model was run separately but are presented together in this table.
Sample includes all patients who met the study inclusion criteria, with follow up as the point of service discharge.

For peer review only

# Estimating the effectiveness of an enhanced 'Improving Access to Psychological Therapies' (IAPT) service addressing the wider determinants of mental health: a real-world evaluation

| | |
|---|---|
| Journal: | *BMJ Open* |
| Manuscript ID | bmjopen-2023-077220.R1 |
| Article Type: | Original research |
| Date Submitted by the Author: | 21-Sep-2023 |
| Complete List of Authors: | Porter, Alice; University of Bristol, Population Health Sciences<br>Franklin, Matthew; The University of Sheffield, Health Economic and Decision Science<br>De Vocht, Frank; University of Bristol, Population Health Sciences; NIHR Applied Research Collaboration West (NIHR ARC West)<br>d'Apice, Katrina; University of Bristol, Population Health Sciences<br>Curtin, Esther; London School of Hygiene & Tropical Medicine, Population Health, Faculty of Epidemiology and Population Health<br>Albers, Patricia ; University of Bristol, Population Health Sciences<br>Kidger, Judi; University of Bristol, Population Health Sciences |
| <b>Primary Subject Heading</b>: | Mental health |
| Secondary Subject Heading: | Health services research, Public health |
| Keywords: | Adult psychiatry < PSYCHIATRY, MENTAL HEALTH, Electronic Health Records |
| | |

SCHOLARONE™ Manuscripts

*I, the Submitting Author has the right to grant and does grant on behalf of all authors of the Work (as defined in the below author licence), an exclusive licence and/or a non-exclusive licence for contributions from authors who are: i) UK Crown employees; ii) where BMJ has agreed a CC-BY licence shall apply, and/or iii) in accordance with the terms applicable for US Federal Government officers or employees acting as part of their official duties; on a worldwide, perpetual, irrevocable, royalty-free basis to BMJ Publishing Group Ltd ("BMJ") its licensees and where the relevant Journal is co-owned by BMJ to the co-owners of the Journal, to publish the Work in this journal and any other BMJ products and to exploit all rights, as set out in our licence.*

*The Submitting Author accepts and understands that any supply made under these terms is made by BMJ to the Submitting Author unless you are acting as an employee on behalf of your employer or a postgraduate student of an affiliated institution which is paying any applicable article publishing charge ("APC") for Open Access articles. Where the Submitting Author wishes to make the Work available on an Open Access basis (and intends to pay the relevant APC), the terms of reuse of such Open Access shall be governed by a Creative Commons licence – details of these licences and which Creative Commons licence will apply to this Work are set out in our licence referred to above.*

*Other than as permitted in any relevant BMJ Author's Self Archiving Policies, I confirm this Work has not been accepted for publication elsewhere, is not being considered for publication elsewhere and does not duplicate material already published. I confirm all authors consent to publication of this Work and authorise the granting of this licence.*

For peer review only

# Estimating the effectiveness of an enhanced 'Improving Access to Psychological Therapies' (IAPT) service addressing the wider determinants of mental health: a real-world evaluation

Alice Porter[1], Matthew Franklin[2], Frank de Vocht[1,3], Katrina d'Apice[1], Esther Curtin[4], Patricia N Albers[1], Judi Kidger[1]

[1] Centre for Public Health, Population Health Sciences, Bristol Medical School, University of Bristol, UK

[2] School for Health and Related Research (ScHARR), University of Sheffield, UK

[3] NIHR Applied Research Collaboration West (NIHR ARC West), Bristol, UK

[4] Department of Population Health, Faculty of Epidemiology and Population Health, London School of Hygiene and Tropical Medicine, UK

Key words: IAPT, mental health, evaluation study

**Correspondence to:**

**Alice Porter,** Centre for Public Health, Population Health Sciences, Bristol Medical School, Canynge Hall, 39 Whatley Road, Bristol, BS8 2PS, UK

Alice.porter@bristol.ac.uk

Word count: 3959

Contributors: JK, FDV, and MF were responsible for study conceptualisation and funding acquisition, AP, EC, KDA and PA were responsible for project administration, PA, KDA, FDV, MF, AP and JK contributed to methodology and the analysis plan, AP and MF conducted the analyses. AP wrote the first draft of the manuscript. All authors contributed to and approved the final manuscript.

Funding: This study was supported by the National Institute for Health and Care Research (NIHR) School for Public Health Research (SPHR), Grant Reference Number SPHR-PHPES004-IAPT. The views expressed are those of the author(s) and not necessarily those of the NIHR or the Department of Health and Social Care.

For peer review only



For peer review only - http://bmjopen.bmj.com/site/about/guidelines.xhtml

## ABSTRACT

**Background.** Addressing the wider determinants of mental health alongside psychological therapy could improve mental health service outcomes and population mental health.

**Objectives.** To estimate the effectiveness of an enhanced 'Improving Access to Psychological Therapies' (IAPT) mental health service compared to traditional IAPT in England. Alongside traditional therapy treatment, the enhanced service included wellbeing support and community service links.

**Design:** A real-world evaluation using IAPT's electronic health records.

**Setting:** Three NHS IAPT services in England**.**

**Participants:** Data from 17,642 service users classified as having a case of depression and/or anxiety at baseline.

**Intervention:** We compared the enhanced IAPT service (intervention) to an IAPT service in a different region providing traditional treatment only (geographical-control), and the IAPT service with traditional treatment before additional support was introduced (historical-control).

**Primary outcome measures:** Patient Health Questionnaire-9 (PHQ-9) depression scale (score range: 0-27) and Generalised Anxiety Disorder-7 (GAD-7) anxiety scale (score range: 0-21); for both, lower scores indicate better mental health. Propensity scores were used to estimate inverse probability of treatment weights, subsequently used in mixed-effects regression models.

**Results:** Small improvements (mean, 95% CI) were observed for PHQ-9 (depression) (-0.21, -0.32 to -0.09) and GAD-7 (anxiety) (-0.23, -0.34 to -0.13) scores in the intervention-group compared to the historical-control. There was little evidence of statistically significant differences between intervention and geographical-control.

**Conclusions:** Embedding additional health and wellbeing support into standard IAPT services may lead to improved mental health outcomes. However, the lack of improved outcomes compared to the geographical-control may instead reflect a more general improvement to the intervention IAPT service. It is not clear from our findings whether an IAPT service with additional health and wellbeing support is clinically superior to traditional IAPT models.




For peer review only - http://bmjopen.bmj.com/site/about/guidelines.xhtml

## Strengths and limitations of this study

- This study is the first to estimate the effectiveness of embedding health and wellbeing support into the traditional NHS mental health service in England.
- This study utilised real-world electronic health record data to explore the effectiveness of an enhanced Improving Access to Talking Therapies (IAPT) service.
- Due to the nature of the research, this study was non-randomised and involved comparing the enhanced IAPT service (intervention) to the existing service (historical-control), whereby potential changes to the service over time could not be accounted for.
- Outcome measures were guided by IAPT's key performance indicators and electronic health records. However, no data was available on the wider determinants of mental health, such as wellbeing and quality of life.

For peer review only



For peer review only - http://bmjopen.bmj.com/site/about/guidelines.xhtml

## INTRODUCTION

Approximately one in six adults in England have a diagnosable mental health disorder (MHD), for which prevalence and severity has worsened over the last 30 years, exacerbated by the COVID-19 pandemic.(1, 2) Preventing and treating MHDs effectively is a global public health priority.

Depression and anxiety are globally considered common MHDs, due to their prevalence.(3) Evidence-based effective treatment for common MHDs includes psychological therapies, e.g. cognitive behaviour therapy (CBT) or psychotherapy.(4) The National Health Service (NHS) England's *Improving Access to Psychological Therapies* (IAPT) programme provides evidence-based psychological therapies for MHDs. IAPT treatment starts with a GP referral or self-referral, with subsequent initial assessment by IAPT staff for anxiety and/or depression 'caseness', followed by allocation to the waiting-list before the first treatment session can be provided. Those requiring IAPT treatment are initially offered a multi-session course with a Psychological Wellbeing Practitioner (PWP), dependent on their symptom severity.(5)

IAPT defines anxiety and depression caseness as a service-user reported score of ≥8 on the Generalised Anxiety Disorder-7 questionnaire (GAD-7) and ≥10 on the Patient Health Questionnaire (PHQ-9), respectively. IAPT's patient-reported key performance indicators (KPIs) are based on the service-users' first (i.e. initial assessment) and last (i.e. at discharge) PHQ-9 and GAD-7 scores; this includes:

- *Recovery*: moving from 'caseness' (PHQ-9 ≥10; GAD-7 ≥8) on either measure to 'no caseness' (PHQ-9 <10; GAD-7 <8) on both measures.
- *Reliable change*: decrease of ≥6 on PHQ-9 or ≥4 on GAD-7, without score increase of PHQ-9 ≥6 or GAD-7 ≥4 on the other measure.
- *Reliable recovery*: achieving both recovery and reliable change.

Of 1.81 million adults referred to IAPT in 2021/22, approximately 50% were defined as 'recovered' at the point of service discharge.(5) However, a large proportion of people do not show reliable change or recover by the point of IAPT discharge. Additionally, common MHD prevalence has not reduced since the IAPT programme started in 2008.(6)

Evidence suggests factors such as unemployment, poverty, debt, abuse, social isolation, poor health and wellbeing and physical inactivity can cause and/or worsen mental health conditions(3) and therefore increase the need for services such as IAPT. The World Health



For peer review only - http://bmjopen.bmj.com/site/about/guidelines.xhtml

Organisation (WHO) suggests mental health services should consider these psychosocial factors when providing treatment.(3) To address this, a ‘whole person’ approach to mental health is required, including strengthening the links between IAPT and wider community and wellbeing support.(7, 8) Focusing on these wider determinants could help to improve the likelihood of recovery from mental health issues by addressing external factors causing these issues. A randomised controlled trial (RCT) found social prescribing (i.e., making referrals from a primary health care service to a voluntary community service) reduced anxiety and improved quality-of-life, but cost more and did not reduce depression severity.(9) An evaluation of IAPT linked with employment services reported that service-users who accessed employment support had better employment-related outcomes, with some indication of mental health improvements.(10)

As part of the mixed-methods ‘Assessing a Distinct Improving Access to Psychological Therapies service’ (ADAPT) study, we aimed to evaluate a new, locally enhanced IAPT service model, which introduced an additional health and wellbeing (H&W) pathway to address wider determinants of mental health. The aim of the quantitative evaluation reported in this article was to estimate and report the effectiveness of the enhanced IAPT service compared to IAPT Treatment-As-Usual (TAU). We hypothesise that the enhanced IAPT service will lead to improved mental health outcomes and waiting times, due to its’ ability to address the wider determinants of mental health, as well as provide treatment for MHDs.

## METHODS

### Intervention

The enhanced IAPT service (intervention) was introduced in March 2021 in three local authorities in South West (SW) England and replaced the existing IAPT service. The enhanced IAPT service provides a more tailored and holistic treatment plan, whereby after an initial assessment with a PWP at baseline (i.e. before waiting-list allocation), service-users can be referred to IAPT TAU only (e.g., psychotherapy), IAPT TAU plus H&W pathway, or the H&W pathway alone. During the initial assessment, PWPs will seek to understand any external causes for a service users’ anxiety or depression. For example, a service user presenting with anxiety due to large amounts of debt may be offered therapy to address their anxiety and be referred to debt management via the H&W pathway, whereas a service user with a more general form of anxiety may only be offered therapy. Therefore, the intervention



For peer review only - http://bmjopen.bmj.com/site/about/guidelines.xhtml

group is made up of service users receiving any of these three treatment options within the enhanced IAPT service in SW England.

The H&W pathway consisted of two elements. First, the 'Healthy Living Healthy Minds' programme, a six-session group webinar series offering guided exercise and advice on healthy lifestyles. Secondly, and/or, one-to-one sessions with a 'Wellbeing Navigator', who facilitated access to community organisations to address the wider psychosocial problems individuals were experiencing (e.g., poverty, unemployment, and social isolation). Supplementary Figure 1 illustrates the enhanced IAPT service pathways. Further details of the H&W pathway are described in the qualitative evaluation.(11)

**Study design and Target Trial protocol**

This was a service-led public health evaluation. A non-randomised study(12, 13) was conducted using IAPT's electronic health records (EHRs). Treatment allocation (at service-level) was non-randomised because it was not possible for the researchers to control its allocation, as the introduction of the enhanced IAPT service resulted from a new service provider and service redesign rather than a research study design (e.g. RCT). The enhanced IAPT service (intervention) was compared to the previous IAPT service with TAU (historical-control), and to a standard IAPT service with TAU in South-East England (geographical-control). The historical-control was provided by a different service provider and the geographical-control by the same service provider as the intervention.

Table 1 provides an overview of the *a-priori* specified Target Trial (TT) framework protocol, which aims to reduce bias by clearly articulating seven study dimensions: eligibility criteria, treatment strategies, assignment procedure, follow-up period, outcomes, estimand(s), analysis plan.(13) The TT framework is intended to mimic the ideal 'pragmatic' RCT.

The Health Research Authority and Health and Care Research Wales granted ethical approval (reference number: 21/PR/0230). The study's planning, conducting and reporting follows the National Institute for Health and Care Excellence (NICE) Real-World Evidence (RWE) framework.(14) The RECORD-PE checklist is provided in the Supplementary Table 1.(15)



For peer review only - http://bmjopen.bmj.com/site/about/guidelines.xhtml

**Table 1. Target Trial protocol summary**

| Component | Description |
|---|---|
| **Eligibility criteria** | **New referrals to IAPT:** no attendance at the IAPT site in the previous 6-months since the new referral<br>**Newly referred during:** March 2021 to March 2022 (intervention & geographical control) or March 2018 to March 2019 (historical control)<br>**Condition caseness at baseline:** service-users classified as having depression caseness (PHQ-9≥10) or anxiety caseness (i.e. GAD-7≥8) at baseline (i.e. before waiting-list allocation)<br>**Baseline data:** recorded PHQ-9 (depression severity) and GAD-7 (anxiety severity) score at baseline – see '**Condition caseness at baseline**'<br>**As-started treatment:** service-users had attended at least one treatment session to be defined as 'as-started' treatment |
| **Treatment strategies** | **Intervention:** Enhanced IAPT service (South West, England), as treatment-as-usual (TAU) plus 'Healthy Living Healthy Minds' programme and/or 1:1 Wellbeing Navigator sessions.<br>**Geographical control:** TAU IAPT service in South East, England;<br>**Historical control:** TAU IAPT service in the intervention area but before the enhanced service had been implemented. |
| **Assignment procedures** | **Non-randomised and unblinded:** referrals are assessed for eligibility by the service, if deemed eligible are offered TAU (across all groups) or TAU plus 'Healthy Living Healthy Minds' programme and/or 1:1 Wellbeing Navigator sessions (intervention only), with uptake based on service-user preference. |
| **Follow-up period(s)** | Starts at baseline appointment to assess condition caseness and allocate people to the waiting-list before first treatment session, and ends at discharge from service or self-discharge from service. |
| **Outcome(s)** | **Primary:** PHQ-9 score (continuous), GAD-7 score (continuous), and WSAS score (continuous).<br>**Secondary:** reliable change (binary), recovery (binary variables), and waiting times (continuous variable). |
| **Estimand(s) (casual contrasts)** | **As-started primary:** in new referrals to IAPT, the between-group difference in mean PHQ-9 or GAD-7 or WSAS for those referred to the enhanced IAPT service compared to IAPT treatment-as-usual in the geographical or historical control at the point of service discharge, regardless of TAU received<br>**Sensitivity analyses:** in new referrals to IAPT, what is the between-group difference in mean PHQ-9 or GAD-7 or WSAS for those who have been within the service for at least 12 or 16 or 20 or 24 weeks, referred to the enhanced IAPT service compared to IAPT treatment-as-usual in the geographical or historical control at the point of service discharge, regardless of TAU received |
| **Analysis plan** | **Overall:** An analytical plan was pre-specified.<br>**Propensity scores:** logistic regression estimated propensity scores were used to derive inverse probability of treatment weights (IPTWs)<br>**Weighted regressions:** Linear mixed effects weighted-regression analyses were conducted for primary outcomes, with the weights based on IPTWs. Linear and logistic regression analyses were conducted for secondary outcomes. Unadjusted, fully adjusted and doubly robust (fully adjusted and weighted) model results were compared for each outcome.<br>**Sub-groups:** those within the intervention group who accessed: (1) the IAPT TAU only; (2) IAPT TAU plus the enhanced service (N.B. no service-users were offered the enhanced service without TAU).<br>**Health inequalities:** the primary outcome models were re-run with additional interactions between treatment group and age, gender, ethnicity, or IMD. |

Abbreviations: IAPT; Improving Access to Psychological Therapies, TAU; treatment-as-usual, PHQ-9; Patient Health Questionnaire-9, GAD-7; Generalized Anxiety Disorder questionnaire-7, WSAS; Work and Social Adjustment Scale, N.B.; nota bene (i.e. please note), IMD; Index of Multiple Deprivation

For peer review only



For peer review only - http://bmjopen.bmj.com/site/about/guidelines.xhtml

## Measures

We used IAPT's EHR data, anonymised before sharing (i.e. on IAPT premises) in line with information governance processes for secondary data analysis. Demographics (age, sex, ethnicity, postcode, and employment status) were provided as baseline variables. Postcodes were matched to a national Index of Multiple Deprivation (IMD) dataset, as an indicator of socioeconomic status (SES).(16) Primary outcomes included service-user reported scores as continuous variables:

- PHQ-9 depression score from 0 (best state) to 27 (worst state)
- GAD-7 anxiety scores from 0 (best state) to 21 (worst state)
- Work and Social Adjustment (WSAS) scale from 0 (best state) to 40 (worst state).

Secondary outcomes included KPIs reliable change (binary), recovery (binary) and waiting time (continuous). *Reliable change* and *recovery* (based on PHQ-9 and GAD-7 scores) were calculated and reported only for those who reached the point of service discharge, in line with the NHS England's IAPT manual(4). Waiting time was defined as the duration in days from referral to first appointment, as reported in the EHRs.

## Eligible participants

Eligible participants were referrals to IAPT classified as having depression (PHQ-9) and/or anxiety (GAD-7) 'caseness' at baseline, with 'baseline' defined as the initial assessment before waiting-list allocation, as part of TAU. For analyses focused on WSAS, eligible participants must have completed WSAS at baseline.

Intervention and geographical-controls were service-users in the relevant area who were referred between March 2021 and March 2022. Historical controls were service-users in the intervention area, referred between March 2018 and March 2019 (i.e. before the enhanced service was introduced when the service was just IAPT TAU); data from service-users referred between April 2019 and February 2021 were not requested, to minimise spill-over effects from the historical-control into the intervention group. All eligible service-users had to have completed at least one treatment session to be included in the analyses.

## Statistical analyses

Descriptive and statistical analysis was conducted using Stata 15. Data preparation included recoding and deriving variables, and merging datasets required for analysis.



For peer review only - http://bmjopen.bmj.com/site/about/guidelines.xhtml

Propensity scores were estimated using logistic regression with treatment assignment as the dependent variable and clinical (baseline PHQ-9, GAD-7 and WSAS) and demographic variables (age, sex, IMD and ethnicity) as independent variables. The propensity scores were used to calculate inverse probability of treatment weights (IPTWs) used within mixed-effects regression models as a 'doubly robust' (i.e., weighted-regression) approach for balancing the comparison groups with respect to baseline covariates. This approach is defined as 'doubly robust' because combining the IPTWs and regression methods means only the weights or the regression need be correctly specified to obtain an unbiased effect estimator; thus minimising between-group baseline imbalances while accounting for baseline confounding due to the non-randomised treatment allocation.(17) Employment status was not included in the regression models due to the proportion of missing data (19%). Data were visually inspected to check the level of balance between groups after reweighting (see Supplementary Figure 2).

Linear mixed-effects weighted-regression analyses were conducted for the primary outcomes, to account for repeated measures, with service-user ID as the random effects variable. For the primary outcomes, a negative average treatment effect on the treated (ATT) represents an improvement to mental health. Linear and logistic weighted-regressions were conducted for secondary outcomes. Model residuals were inspected to test assumptions of distribution, linearity, and homoscedasticity. A complete-case analysis was conducted by focusing the analysis on people with at least one follow-up data collection time-point, thus assuming missing outcome scores were missing-completely-at-random. The follow-up period started at baseline and ended at discharge from the service, to align with IAPT's KPIs.(4)

**Sensitivity and sub-group analyses**

Sensitivity analyses were conducted to explore outcomes among samples of service users who had been within the service for at least 12, 16, 20 and 24-weeks, respectively, with the follow-up period ending at discharge from the service. These analyses were based on evidence suggesting at least 8-12 weeks of treatment is required to observe clinical change in mental health disorders.(18-20)

Sub-group analyses included those within the intervention group who accessed: 1) the IAPT TAU only; 2) IAPT TAU plus H&W pathway (NB: no service-users were offered the H&W pathway without TAU).



For peer review only - http://bmjopen.bmj.com/site/about/guidelines.xhtml

To explore potential health inequalities, the primary outcome models were re-run with additional interactions between treatment-group and age, sex, ethnicity, or IMD. Effect sizes, 95% confidence intervals (CI) and p-values were reported.

## Patient and Public Involvement

The development of the research proposal was overseen by a local IAPT steering group. The service provider and service users were involved in dissemination plans of the research.

## RESULTS

### Descriptive statistics

Table 2 presents demographic characteristics of the eligible participants, which were similar across intervention and control groups. Supplementary Table 2 presents the number of service-users with data available in the IAPT EHRs, who met study eligibility criteria, and who were included in analyses.

Table 2 also presents the mental health outcomes descriptively, showing that the mean baseline mental health scores were 1-2 points lower in the intervention vs control groups. The percentage of service-users achieving PHQ-9 reliable change were similar for intervention and historical-control (both 43%), but higher for geographical-controls (50%). Percentage of service-users achieving GAD-7 reliable change: intervention, 56%; geographical, 58%; historical, 52%. The percentage who recovered was similar for intervention and geographical-control (both 48%), but lower for historical-controls (38%).



For peer review only - http://bmjopen.bmj.com/site/about/guidelines.xhtml

**Table 2. Descriptive statistics for demographics and mental health measures scores for eligible sample**

| | | Intervention | | | | | | | | | Geographical | | | Historical | | |
|---|---|---|---|---|---|---|---|---|---|---|---|---|---|---|---|---|
| | | Intervention | | | IAPT TAU + H&W | | | IAPT TAU only | | | | | | | | |
| ***Measure*** | ***Sub-measure*** | ***N*** | ***%*** | | ***N*** | ***%*** | | ***N*** | ***%*** | | ***N*** | ***%*** | | ***N*** | ***%*** | |
| N overall | N/A | 8,020 | 100 | | 728 | 100 | | 7292 | 100 | | 3,548 | 100 | | 6,074 | 100 | |
| Sex | Female | 5,558 | 69 | | 494 | 68 | | 5,064 | 69 | | 2,461 | 69 | | 3,948 | 65 | |
| Ethnicity | Asian | 223 | 3 | | 30 | 4 | | 193 | 3 | | 105 | 3 | | 183 | 3 | |
| | Black | 175 | 2 | | 29 | 4 | | 146 | 2 | | 121 | 3 | | 175 | 3 | |
| | Mixed | 271 | 3 | | 26 | 4 | | 245 | 3 | | 88 | 2 | | 192 | 3 | |
| | Other | 100 | 1 | | 13 | 2 | | 87 | 1 | | 23 | 1 | | 62 | 1 | |
| | White | 7,109 | 89 | | 610 | 84 | | 6,499 | 89 | | 3,132 | 88 | | 5,336 | 88 | |
| Employment | Employed | 4,633 | 58 | | 382 | 52 | | 4,251 | 58 | | 1,812 | 51 | | 3,537 | 58 | |
| | Unemployed | 923 | 12 | | 163 | 22 | | 760 | 10 | | 758 | 21 | | 1,156 | 19 | |
| | Retired | 261 | 3 | | 37 | 5 | | 224 | 3 | | 235 | 7 | | 291 | 5 | |
| | Student | 679 | 8 | | 35 | 5 | | 644 | 9 | | 141 | 4 | | 397 | 7 | |
| PHQ-9 RC [a] | No | 3,346 | 57 | | 414 | 62 | | 2932 | 57 | | 1467 | 50 | | 3096 | 58 | |
| | Yes | 2,507 | 43 | | 256 | 38 | | 2251 | 43 | | 1449 | 50 | | 2276 | 42 | |
| GAD-7 RC [a] | No | 2,549 | 44 | | 350 | 52 | | 2199 | 42 | | 1226 | 42 | | 2594 | 48 | |
| | Yes | 3,304 | 56 | | 320 | 48 | | 2984 | 58 | | 1690 | 58 | | 2778 | 52 | |
| Recovered [a] | No | 3019 | 52 | | 485 | 72 | | 2534 | 49 | | 1507 | 52 | | 3342 | 62 | |
| | Yes | 2834 | 48 | | 185 | 28 | | 2649 | 51 | | 1409 | 48 | | 2030 | 38 | |
| ***Measure*** | ***Sub-measure*** | ***Mean*** | ***SD*** | ***Range*** | ***Mean*** | ***SD*** | ***Range*** | ***Mean*** | ***SD*** | ***Range*** | ***Mean*** | ***SD*** | ***Range*** | ***Mean*** | ***SD*** | ***Range*** |
| Age | N/A | 35 | 14 | 15-92 | 40 | 15 | 16-89 | 35 | 13 | 15-92 | 39 | 16 | 15-107 | 37 | 14 | 16-93 |
| IMD | Decile | 5.5 | 1.9 | - | 5.3 | 1.9 | - | 5.5 | 1.9 | - | 5.7 | 2.3 | - | 5.2 | 1.8 | 2.4-9.1 |
| PHQ-9 | Baseline | 15 | 5 | 0-27 | 17 | 5 | 1-27 | 14 | 5 | 0-27 | 16 | 5 | 0-27 | 16 | 5 | 0-27 |
| | Last obs. | 11 | 6 | 0-27 | 13 | 7 | 0-27 | 10 | 6 | 0-27 | 11 | 7 | 0-27 | 11 | 7 | 0-27 |
| | Dif. | -4.1 | 5.7 | -26 - 22 | -4.0 | 5.9 | -25 - 18 | -4.1 | 5.7 | -26 - 22 | -5.2 | 6.3 | -27 - 15 | -4.4 | 6 | -26 - 20 |
| GAD-7 | Baseline | 13 | 4 | 0-21 | 14 | 5 | 0-21 | 13 | 4 | 0-21 | 14 | 4 | 0-21 | 14 | 4 | 0-21 |
| | Last obs. | 9 | 6 | 0-21 | 11 | 6 | 0-21 | 9 | 6 | 0-21 | 10 | 6 | 0-21 | 10 | 6 | 0-21 |
| | Dif. | -3.9 | 5.4 | -21 - 17 | -3.4 | 5.3 | -21 - 14 | -4.0 | 5.4 | -21 - 17 | -4.7 | 5.8 | -21 - 14 | -3.9 | 5.7 | -21 - 19 |
| WSAS | Baseline | 18 | 8 | 0-40 | 21 | 9 | 0-40 | 18 | 8 | 0-40 | 19 | 9 | 0-40 | 20 | 9 | 0-40 |
| | Last obs. | 15 | 9 | 0-40 | 18 | 10 | 0-40 | 14 | 9 | 0-40 | 15 | 10 | 0-40 | 16 | 10 | 0-40 |
| | Dif. | -3.2 | 8.4 | -38 - 37 | -3.0 | 9.1 | -28 - 30 | -3.2 | 8.3 | -38 - 37 | -4.3 | 9.3 | -40 - 36 | -4.4 | 9.6 | -39 - 40 |
| Waiting time (days) | To 1st app. | 20 | 25 | 0-336 | 19 | 21 | 0-240 | 20 | 26 | 0-336 | 10 | 20 | 0 - 322 | 20 | 18 | 0-216 |
| No. sessions attended | To discharge | 5 | 3 | 1-32 | 8 | 6 | 1-32 | 5 | 3 | 1-27 | 5 | 3 | 1-25 | 6 | 4 | 1-30 |

Abbreviations: TAU; treatment-as-usual, H&W; Health & Wellbeing pathway, IMD; Index of Multiple Deprivation, SD; standard deviation PHQ-9; Physical Health Questionnaire-Depression Scale, GAD-7; Generalised Anxiety Disorder Questionnaire 7, WSAS; Work and Social Adjustment Scale, RC; Reliable change (defined as the reduction of six points or more on the PHQ-9 or four points or more on the GAD-7). Recovered, defined as moving below the threshold of ten on PHQ-9 or eight on GAD-7

[a] Includes patients who have been discharged from the service only.



For peer review only - http://bmjopen.bmj.com/site/about/guidelines.xhtml

**Primary analyses**

Table 3 indicates there was little difference in the ATT for PHQ-9, GAD-7 and WSAS when comparing the intervention to geographical-control. Compared to the historical-control, we observed a small, negative ATT for PHQ-9 (-0.40, 95% CI -0.53, -0.27), GAD-7 (-0.43, 95% CI -0.55, -0.32) and WSAS (-0.53, 95% CI -0.53, -0.15), representing small improvements in mental health in the intervention.

Table 3 indicates the odds of achieving PHQ-9 or GAD-7 reliable change were similar between intervention vs geographical-control. The odds of recovery were lower in the intervention vs geographical-control (OR=0.87, 95% CI 0.78, 0.96). Conversely, the odds were higher in the intervention vs historical-control for achieving PHQ-9 reliable change (OR=1.22, 95% CI 1.12, 1.33), GAD-7 reliable change (OR=1.17, 95% CI 1.17, 1.38), and recovery (OR=1.46, 95% CI 1.34, 1.59). Waiting-times were longer in the intervention vs geographical-control (mean = 10 days, 95% CI 9.21, 11.52) and marginally longer vs historical-control (mean = 1.5 days, 95% 0.67, 2.41).



For peer review only - http://bmjopen.bmj.com/site/about/guidelines.xhtml

**Table 3. Doubly robust mixed effects linear regression models for primary and secondary outcomes in as-started sample**

| | Intervention vs Geographical Control n obs = 48,652, n patients = 8,542 | | | | Intervention vs Historical Control n obs = 64,044, n patients = 10,951 | | | |
|---|---|---|---|---|---|---|---|---|
| | **Coefficient** | **95% CI** | | **p value** | **Coefficient** | **95% CI** | | **p value** |
| PHQ-9 | -0.08 | -0.23 | 0.07 | 0.284 | -0.40 | -0.53 | -0.27 | <0.001 |
| GAD-7 | -0.13 | -0.27 | 0.00 | 0.052 | -0.43 | -0.55 | -0.32 | <0.001 |
| WSAS | 0.09 | -0.14 | 0.32 | 0.437 | -0.34 | -0.53 | -0.15 | <0.001 |
| PHQ-9 reliable change | 0.90 | 0.82 | 1.00 | 0.045 | 1.22 | 1.12 | 1.33 | <0.001 |
| GAD-7 reliable change | 1.00 | 0.90 | 1.10 | 0.941 | 1.27 | 1.17 | 1.38 | <0.001 |
| Recovery | 0.87 | 0.78 | 0.96 | 0.007 | 1.46 | 1.34 | 1.59 | <0.001 |
| Waiting times | 10.37 | 9.21 | 11.52 | <0.001 | 1.54 | 0.67 | 2.41 | 0.001 |

All models are adjusted for covariates (age, gender, ethnicity, IMD, baseline PHQ-9, baseline GAD-7 and baseline WSAS). Inverse propensity score weighting applied to all models, weighted for all covariates.
Sample includes all patients who met the study inclusion criteria, with follow up as the point of service discharge.
Reliable change PHQ-9 is defined as a reduction of ≥6 points. For patients to have a measure of reliable change PHQ-9 they must have been discharged.
Reliable change GAD-7 is defined as a reduction of ≥4 points. For patients to have a measure of reliable change GAD-7 they must have been discharged.
Recovery is defined as entering the service at the threshold of a score of ≥10 on PHQ-9 and/or ≥8 on GAD-7 and moving to "no-caseness", being a score below the threshold on both measures of PHQ-9 and GAD-7. For a patient to have a measure of recovery they must have been discharged.

For peer review only



For peer review only - http://bmjopen.bmj.com/site/about/guidelines.xhtml

## Sensitivity analyses

Table 4 presents the sensitivity analysis results, which indicate no difference in ATT between intervention and geographical-control, except for a small negative ATT (i.e. improved score) for GAD-7 in the 24-week sample (-0.22, 95% CI -0.39, -0.05). Comparing the intervention to historical-control, small negative ATTs (i.e. improved scores) were observed for PHQ-9, GAD-7 and WSAS across all four samples, although associations were weak for WSAS in the 16-week to 24-week samples.

The odds of achieving PHQ-9 reliable change were higher in the intervention group vs geographical-control in the 24-week sample only (OR=1.15, 95% CI 1.02, 1.30). The odds of achieving GAD-7 reliable change were higher in the intervention group vs geographical control in the 20-week (OR=1.17, 95% CI 1.04, 1.32) and 24-week samples (OR=1.20, 95% CI 1.05, 1.36). The odds of recovery were not different between intervention and geographical-control. The odds of achieving PHQ-9 reliable change, GAD-7 reliable change and recovery were higher in the intervention vs historical-control across all four samples.

Across the four samples, findings suggest 10-12 days longer waiting-times in the intervention group compared to the geographical control group. Waiting times were slightly longer by 1.6-3.0 days in the intervention group compared to historical-control in the 16-week to 24-week samples, but no difference was observed in the 12-week sample.



For peer review only - http://bmjopen.bmj.com/site/about/guidelines.xhtml

**Table 4. Doubly robust mixed effects linear regression models for primary and secondary outcomes for patients within the service for at least 12-24 weeks**

| Measure | 12-weeks | | | | 16-weeks | | | | 20-weeks | | | | 24-weeks | | | |
|---|---|---|---|---|---|---|---|---|---|---|---|---|---|---|---|---|
| | Coef. | 95% CI | | p value | Coef. | 95% CI | | p value | Coef. | 95% CI | | p value | Coef. | 95% CI | | p value |
| | **Intervention vs Geographical Control** | | | | | | | | | | | | | | | |
| PHQ-9 | -0.11 | -0.29 | 0.07 | 0.229 | -0.13 | -0.31 | 0.04 | 0.131 | -0.10 | -0.28 | 0.08 | 0.268 | -0.19 | -0.38 | 0.01 | 0.056 |
| GAD-7 | -0.10 | -0.26 | 0.06 | 0.216 | -0.12 | -0.28 | 0.04 | 0.127 | -0.12 | -0.29 | 0.04 | 0.143 | -0.22 | -0.39 | -0.05 | 0.012 |
| WSAS | 0.20 | -0.07 | 0.46 | 0.141 | 0.03 | -0.24 | 0.29 | 0.833 | 0.00 | -0.27 | 0.28 | 0.978 | -0.10 | -0.39 | 0.18 | 0.476 |
| PHQ-9 RC | 1.05 | 0.93 | 1.18 | 0.459 | 1.06 | 0.94 | 1.19 | 0.351 | 1.12 | 0.99 | 1.26 | 0.062 | 1.15 | 1.02 | 1.30 | 0.027 |
| GAD-7 RC | 1.09 | 0.97 | 1.23 | 0.156 | 1.11 | 0.99 | 1.25 | 0.085 | 1.17 | 1.04 | 1.32 | 0.012 | 1.20 | 1.05 | 1.36 | 0.005 |
| Recovery | 1.07 | 0.94 | 1.21 | 0.288 | 1.03 | 0.91 | 1.16 | 0.670 | 1.13 | 1.00 | 1.28 | 0.053 | 1.11 | 0.98 | 1.26 | 0.113 |
| Waiting times | 10.04 | 9.03 | 11.05 | <0.001 | 10.88 | 9.94 | 11.81 | <0.001 | 11.23 | 10.36 | 12.10 | 0.000 | 11.06 | 10.19 | 11.94 | <0.001 |
| | **Intervention vs Historical Control** | | | | | | | | | | | | | | | |
| PHQ-9 | -0.46 | -0.61 | -0.31 | <0.001 | -0.35 | -0.50 | -0.20 | <0.001 | -0.32 | -0.48 | -0.17 | <0.001 | -0.31 | -0.48 | -0.15 | <0.001 |
| GAD-7 | -0.41 | -0.55 | -0.27 | <0.001 | -0.35 | -0.48 | -0.21 | <0.001 | -0.30 | -0.44 | -0.16 | <0.001 | -0.32 | -0.46 | -0.17 | <0.001 |
| WSAS | -0.39 | -0.61 | -0.17 | 0.001 | -0.27 | -0.49 | -0.05 | 0.019 | -0.25 | -0.48 | -0.02 | 0.036 | -0.25 | -0.50 | -0.01 | 0.043 |
| PHQ-9 RC | 1.26 | 1.14 | 1.40 | <0.001 | 1.16 | 1.05 | 1.28 | 0.003 | 1.13 | 1.02 | 1.24 | 0.018 | 1.11 | 1.00 | 1.23 | 0.045 |
| GAD-7 RC | 1.21 | 1.10 | 1.34 | <0.001 | 1.19 | 1.08 | 1.31 | <0.001 | 1.11 | 1.01 | 1.23 | 0.034 | 1.14 | 1.03 | 1.27 | 0.013 |
| Recovery | 1.47 | 1.33 | 1.64 | <0.001 | 1.34 | 1.22 | 1.49 | <0.001 | 1.34 | 1.21 | 1.49 | <0.001 | 1.30 | 1.17 | 1.45 | <0.001 |
| Waiting times | 0.30 | -0.59 | 1.19 | 0.511 | 1.57 | 0.75 | 2.39 | <0.001 | 2.49 | 1.71 | 3.27 | <0.001 | 3.01 | 2.22 | 3.79 | <0.001 |

Abbreviations: RC; reliable change

Intervention Vs. Geographical, N patient / N obs: 12-weeks, 6,021 / 35,999; 16-weeks, 6,364 / 37,965; 20-weeks, 5,972 / 36,310; 24-weeks, 5,499 / 33,829

Intervention Vs. Historical, N patient / N obs: 12-weeks, 7,805 / 47,512; 16-weeks, 8,076 / 50,071; 20-weeks, 7,603 / 47,835; 24-weeks, 6,880 / 43,873

All models are adjusted for covariates (age, gender, ethnicity, IMD, baseline PHQ-9, baseline GAD-7 and baseline WSAS). Inverse propensity score weighting applied to all models, weighted for all covariates.

Samples include all patients who met the study inclusion criteria and were in the service for at least 12, 16, 20, 24 weeks, respectively, with follow up as the point of service discharge.

Reliable change (RC) PHQ-9 is defined as a reduction of ≥6 points. For patients to have a measure of reliable change PHQ-9 they must have been discharged.

Reliable change (RC) GAD-7 is defined as a reduction of ≥4 points. For patients to have a measure of reliable change GAD-7 they must have been discharged.

Recovery is defined as entering the service at the threshold of a score of ≥10 on PHQ-9 and/or ≥8 on GAD-7 and moving to a "no-caseness", being a score below the threshold on both measures of PHQ-9 and GAD-7. For a patient to have a measure of recovery they must have been discharged.

For peer review only



For peer review only - http://bmjopen.bmj.com/site/about/guidelines.xhtml

**Sub-group analyses and investigating health inequalities**

Table 2 shows that within the intervention group, the percentage of service-users achieving PHQ-9 or GAD-7 reliable change was lower among those who received IAPT TAU plus H&W pathway vs IAPT TAU only. However, Table 2 also shows that those who received IAPT TAU plus H&W pathway had slightly higher mean PHQ-9 and GAD-7 scores at baseline.

Supplementary Table 3 shows a group by gender interaction was observed for PHQ-9 and GAD-7 when comparing the intervention to geographical control, suggesting a slightly greater ATT among males in the geographical-control. A group by IMD interaction was observed for GAD-7 and WSAS when comparing intervention to historical-control, suggesting a slightly greater ATT among service users living in areas of low deprivation in the intervention group.

## DISCUSSION

Using a non-randomised study design and routinely collected 'real-world' IAPT EHR data, this study is the first to evaluate the effectiveness of providing an array of support to address the wider determinants of mental health in addition to psychological therapies. When comparing the intervention to the historical-control, our findings indicate that the enhanced service led to greater (albeit small) improvements in mental health scores and a higher number of service-users recovering from their mental disorder. This aligns with the findings from the qualitative evaluation, which suggests the H&W pathway was perceived by service deliverers and users to have a positive impact on mental health.(11) However, when comparing the intervention to the geographical-control over the same time period, we observed little evidence of improvement.

Previous studies have shown IAPT service adaptation can address harmful health behaviours (e.g., smoking)(21) and help treat other mental health-related issues (e.g., insomnia).(22) Our evaluation(11) provides evidence that IAPT services can be adapted to provide person-centred, tailored links to services, to address the underlying reasons for poor mental health, which meets key policy(7) and WHO recommendations.(3) However, in this study the evidence for an additional benefit over psychological therapy is limited, as we observed small improvements in the historical-control only. As the historical-control service was taken over by a new service provider (the same service provider as the geographical-control), it may be that the standard elements of the intervention service, such as the psychological therapy, had



For peer review only - http://bmjopen.bmj.com/site/about/guidelines.xhtml

been improved by the new service provider, which could explain why we observe mental health improvements in the intervention compared to the historical-control but not the geographical-control. In other words we cannot rule out that the effects seen were due to an improved standard service, rather than the enhanced aspects, especially as recovery rates in the historical control service sample were lower than the national average (38% vs 53%)(5)

The reliable change and recovery results were more pronounced than the ATTs from the continuous outcome measure scores. The odds of achieving reliable change was between 11-27% and achieving recovery was between 30-47% greater in the intervention versus historical-control analyses, while the difference in mean PHQ-9 and GAD-7 scores between groups were minimal. The ATTs we observed were smaller compared to a similar study, which evaluated the effectiveness of additional insomnia support alongside TAU within IAPT on clinical mental health outcomes.(22) Stott et al., (2021)(22) reported ATTs of between -0.78 and -1.30 for PHQ-9, GAD-7 and WSAS (sample size of 1020) compared to our estimates, which were between -0.34 and -0.43. Our findings suggest a large individual variability in treatment effectiveness and may partly explain the lack of average improvement observed at the group level. The reasons for this variability likely go beyond the data collected by IAPT (such as demographics and number of sessions attended), and may include factors such as service-user relationships with practitioners and linked services, and service-users' cognition, attitudes and beliefs.(23) Our qualitative evaluation shed some light by highlighting certain implementation issues, such as service users and wider services expressing they lacked clarity and reasoning from PWPs about why a certain referral pathway was chosen.(11) These findings may help to explain the small ATTs and lack of statistical difference compared to the geographical control.

Previous evidence suggests 8-12 weeks is required to observe clinically meaningful changes for common MHDs.(18-20) However, our sensitivity analyses suggest treatment duration may have to be longer. We observed small but favourable intervention effects for anxiety severity and reliable change compared to the geographical-control among service-users receiving treatment for at least 24-weeks. A longer duration may be required to properly establish service-users' links into community services, and associated additional time from the point of addressing the wider determinants of people's mental health issues to the eventual impact on actual mental health outcome scores (e.g. PHQ-9 and GAD-7 scores).



For peer review only - http://bmjopen.bmj.com/site/about/guidelines.xhtml

Our findings suggest further adaptation or additional resources may be required to address potential health inequalities. Specifically, we found a greater improvement in mental health among service-users in the intervention compared to historical-controls, but only for those from a higher socioeconomic background. Relatedly, a systematic review and meta-analysis suggested socioeconomic deprivation was associated with poorer treatment outcomes for mental health.(24) Future research should explore and address the barriers faced by service-users living in deprived areas in achieving optimal treatment effects.

**Strengths and Limitations**

An RCT was not possible as the enhanced IAPT service was part of a local service redesign, thus treatment allocation could not be controlled by the researchers. Instead, we used a quasi-experimental design based on real-world data (i.e., IAPTs EHRs) to estimate the effectiveness of the enhanced IAPT service. This approach has causal inference implications due to the observational nature of the study, which is more prone to bias (e.g., study-entry selection and confounding bias) than RCTs. However, real-world studies have benefits including representing the effectiveness of the intervention under real-world scenarios and using real-world data, which helps improve the external validity (e.g., generalisability) of the results within the setting of interest e.g. IAPT and NHS England. We used the TT framework (12) and NICE's RWE(14) framework to guide our study design and choice of analytical methods, including how we assessed and accounted for bias due to the non-randomised nature of the study. For example, we used a TT protocol, two control groups, and 'doubly robust' weighted-regression models, all of which are designed to reduce the potential impact of study-entry selection bias and confounding bias. Additionally, conducting pre-specified sensitivity analyses with secondary outcomes and different samples increased our ability to assess result robustness. However, as is common, we use a 'no unmeasured confounders' assumption, thus the potential impact of unmeasured confounding has not been fully assessed.(25)

Two control groups were used, however both have limitations to note: we cannot account for potential changes over time in the historical-control and for potential clustering effects in the geographical-control. Outcome score at service discharge was selected as the follow-up end-point to align with IAPT's KPIs of interest, i.e. based on PHQ-9 and GAD-7 scores at service discharge. This may have resulted in informative censoring because we do not know if and how service users' mental health may have changed after the point of discharge. In addition,



For peer review only - http://bmjopen.bmj.com/site/about/guidelines.xhtml

service-users may have been discharged for reasons other than recovery, which was not indicated in IAPT's EHR. For example, early discharge could be due to mental health improvement, or self-discharge due to service dissatisfaction, both impacting on the service-users' outcome score at point of service discharge and beyond discharge. The use of more complex methods such as G-methods (e.g. inverse probability of censoring weighting) required to account for informative censoring were beyond the scope of this study, given these methods have commonly been applied more to time-to-event analyses related to survival than patient-reported outcome measure scores. The application of such methods to outcome measures is an area for future research.

In terms of data suitability, the IAPT's EHR data were preferable compared to primary data collection as it allowed for a large sample size, quicker evaluation, and minimal participant burden. However, dropping variables and associated participants with too much missing data (e.g., employment status) reduced the study sample size and may have introduced some selection bias if our missing-completely-at-random assumption does not hold. In addition, the EHR data did not include measures such as wellbeing, debt management or social isolation, which may have been useful to better understand the mechanistic impact of the H&W pathway.

Although the intervention group was not necessarily defined by those who received the H&W pathway (i.e. also included those who received therapy only), we should highlight that only 9.1% of the intervention group received H&W support. This may have been because PWPs did not deem patients to require the H&W service. However, it may have also have been due to issues regarding intervention delivery. The enhanced service was newly introduced at the time of study, with the process of establishing links with wider community services still ongoing. This, in addition to the COVID-19 pandemic impacting service delivery, could have reduced the ability to link service-users to other support.(26) This could have reduced estimates of effectiveness and provides further evidence that our results represent a more general improvement to the enhanced service versus historical service rather than a positive effect of the H&W support more specifically.

**Clinical Implications**

There is evidence supporting the role of social prescribing through GPs(9) for individuals with physical and mental co-morbidities.(27) This study provides limited evidence that linking clinical mental health services, like IAPT, to public health community services



For peer review only - http://bmjopen.bmj.com/site/about/guidelines.xhtml

addressing wider determinants of mental health may improve mental health outcomes. Stronger inferences cannot be made as we observed improvements in mental health when comparing the intervention to the historical-control but not to the geographical control. Longer term evaluation may be required to allow the enhanced IAPT service to be fully established for any impact to be clear. Mental health services which embed support to address the wider determinants should also consider including additional measures such as wellbeing and social isolation, in order to better understand the pathway to improved mental health.

## CONCLUSION

This study provides evidence of an improved IAPT service over time. However, evidence is less clear that embedding additional health and wellbeing support leads to improved service-user mental health outcomes. Longer follow-up periods may be required to observe any further mental health improvements gained from health and wellbeing support in addition to psychological therapies.

For peer review only



For peer review only - http://bmjopen.bmj.com/site/about/guidelines.xhtml

441 Acknowledgements: We would like to thank the IAPT service providers for providing
442 anonymised electronic health record data and information about the data used in our analyses.

443 Conflicts of Interest: none

444 Ethical Approval Statement: The Health Research Authority and Health and Care Research
445 Wales granted ethical approval (reference number: 21/PR/0230).

446 Data Sharing: All electronic health record data were provided by Improving Access to
447 Psychological Therapies (IAPT) service providers. The terms of our data agreement with
448 IAPT mean we cannot share these data but data may be requested from the service providers.

449 **REFERENCES**

450 1. McManus S, Bebbington P, Jenkins R, et al. Mental health and wellbeing in England:
451 Adult Psychiatric Morbidity Survey 2014. NatCen Social Research and the Department of
452 Health Sciences, University of Leicester; 2016.
453 2. Daly M, Sutin AR, Robinson E. Longitudinal changes in mental health and the
454 COVID-19 pandemic: evidence from the UK Household Longitudinal Study. Psychol Med.
455 2022;52(13):2549-58.
456 3. World Health Organization. Mental health 2019 [Available from:
457 https://www.who.int/health-topics/mental-health#tab=tab_1.
458 4. NHS England. The NHS Talking Therapies manual. 2023.
459 5. NHS Digital. Psychological Therapies, Annual report on the use of IAPT services,
460 2021-22. 2022.
461 6. Bastiampillai TJ, Allison S, Harford P, et al. Has the UK Improving Access to
462 Psychological Therapies programme and rising antidepressant use had a public health
463 impact? Lancet Psychiatry. 2019;6(3):e8-e9.
464 7. Griffiths S, Foster J, S. S, et al. Mental health's market experiment: Commissioning
465 Psychological Therapies Through Any Qualified Provider. Chester: Centre for Psychological
466 Therapies in Primary Care; 2013.
467 8. NHS England. The Community Mental Health Framework for Adults and Older
468 Adults. 2019.
469 9. Grant C, Goodenough T, Harvey I, et al. A randomised controlled trial and economic
470 evaluation of a referrals facilitator between primary care and the voluntary sector. BMJ.
471 2000;320(7232):419-23.
472 10. Hogarth T, Hasluck C, Gambin L, et al. Evaluation of Employment Advisors in the
473 IAPT Programme. 2013.
474 11. Curtin EL, d'Apice K, Porter A, et al. Perspectives on an enhanced 'Improving Access
475 to Psychological Therapies' (IAPT) service addressing the wider determinants of mental
476 health: a qualitative study. BMC Health Serv Res. 2023;23(1):536.
477 12. de Vocht F, Katikireddi SV, McQuire C, et al. Conceptualising natural and quasi
478 experiments in public health. BMC Med Res Methodol. 2021;21(1):32.
479 13. Hernan MA, Robins JM. Using Big Data to Emulate a Target Trial When a
480 Randomized Trial Is Not Available. Am J Epidemiol. 2016;183(8):758-64.
481 14. NICE. NICE real-world evidence framework. 2022.



For peer review only - http://bmjopen.bmj.com/site/about/guidelines.xhtml

15. Langan SM, Schmidt SA, Wing K, et al. The reporting of studies conducted using observational routinely collected health data statement for pharmacoepidemiology (RECORD-PE). BMJ. 2018;363:k3532.
16. Ministry of Housing Communities & Local Government. English indices of deprivation 2019 2019 [Available from: https://imd-by-geo.opendatacommunities.org/imd/2019/area.
17. Funk MJ, Westreich D, Wiesen C, et al. Doubly robust estimation of causal effects. Am J Epidemiol. 2011;173(7):761-7.
18. NICE. Depression in adults: recognition and management. London; 2009.
19. Ramsberg J, Asseburg C, Henriksson M. Effectiveness and cost-effectiveness of antidepressants in primary care: a multiple treatment comparison meta-analysis and cost-effectiveness model. PLoS One. 2012;7(8):e42003.
20. Annemans L, Brignone M, Druais S, et al. Cost-effectiveness analysis of pharmaceutical treatment options in the first-line management of major depressive disorder in Belgium. Pharmacoeconomics. 2014;32(5):479-93.
21. Taylor GMJ, Sawyer K, Kessler D, et al. Views about integrating smoking cessation treatment within psychological services for patients with common mental illness: A multi-perspective qualitative study. Health Expect. 2021;24(2):411-20.
22. Stott R, Pimm J, Emsley R, et al. Does adjunctive digital CBT for insomnia improve clinical outcomes in an improving access to psychological therapies service? Behav Res Ther. 2021;144:103922.
23. Martin LR, Williams SL, Haskard KB, et al. The challenge of patient adherence. Ther Clin Risk Manag. 2005;1(3):189-99.
24. Finegan M, Firth N, Wojnarowski C, et al. Associations between socioeconomic status and psychological therapy outcomes: A systematic review and meta-analysis. Depress Anxiety. 2018;35(6):560-73.
25. Austin PC. An Introduction to Propensity Score Methods for Reducing the Effects of Confounding in Observational Studies. Multivariate Behav Res. 2011;46(3):399-424.
26. Morris SL, Gibson K, Wildman JM, et al. Social prescribing during the COVID-19 pandemic: a qualitative study of service providers' and clients' experiences. BMC Health Serv Res. 2022;22(1):258.
27. Toffolutti V, Stuckler D, McKee M, et al. The employment and mental health impact of integrated Improving Access to Psychological Therapies: Evidence on secondary health care utilization from a pragmatic trial in three English counties. J Health Serv Res Policy. 2021;26(4):224-33.



For peer review only - http://bmjopen.bmj.com/site/about/guidelines.xhtml

# SUPPLEMENTARY MATERIAL

**Supplementary Figure 1. Diagram illustrating the enhanced Improving Access to Psychological Therapies service structure.**
Grey shapes indicate the four types of support offered after PWP assessment; rectangle boxes indicate activities within IAPT; circles indicate activities outside of IAPT; diamonds indicate ongoing review questions. Abbreviations: GP; General Practitioner, PWP; Psychological Wellbeing Practitioner, H&W; Health and Wellbeing Pathway, HLHM; Healthy Living Healthy Minds programme.

**Supplementary Figure 2. Example box plots exploring the balance of covariates for age and baseline GAD-7 after propensity score estimation**

**Supplementary Table 1. The RECORD statement for pharmacoepidemiology (RECORD-PE) checklist of items, extended from the STROBE and RECORD statements, which should be reported in non-interventional pharmacoepidemiologic studies using routinely collected health data**
Abbreviations: RECORD=reporting of studies conducted using observational routinely collected data; RECORD-PE=RECORD for pharmacoepidemiologic research; STROBE=strengthening the reporting of observational studies in epidemiology.

**Supplementary Table 2. Number of service-users included in analyses**

**Supplementary Table 3. Doubly robust mixed effects linear regression models exploring group by demographic interactions effects for primary outcome in as-started sample**
Abbreviations: TAU; treatment-as-usual, H&W; Health & Wellbeing pathway, IMD; Index of Multiple Deprivation, SD; standard deviation PHQ-9; Physical Health Questionnaire-Depression Scale, GAD-7; Generalised Anxiety Disorder Questionnaire 7, WSAS; Work and Social Adjustment Scale, RC; Reliable change (defined as the reduction of six points or more on the PHQ-9 or four points or more on the GAD-7). Recovered, defined as moving below the threshold of ten on PHQ-9 or eight on GAD-7
[a] Includes patients who have been discharged from the service only.

For peer review only

**Supplementary Figure 1. Diagram illustrating the enhanced Improving Access to Psychological Therapies service structure.**

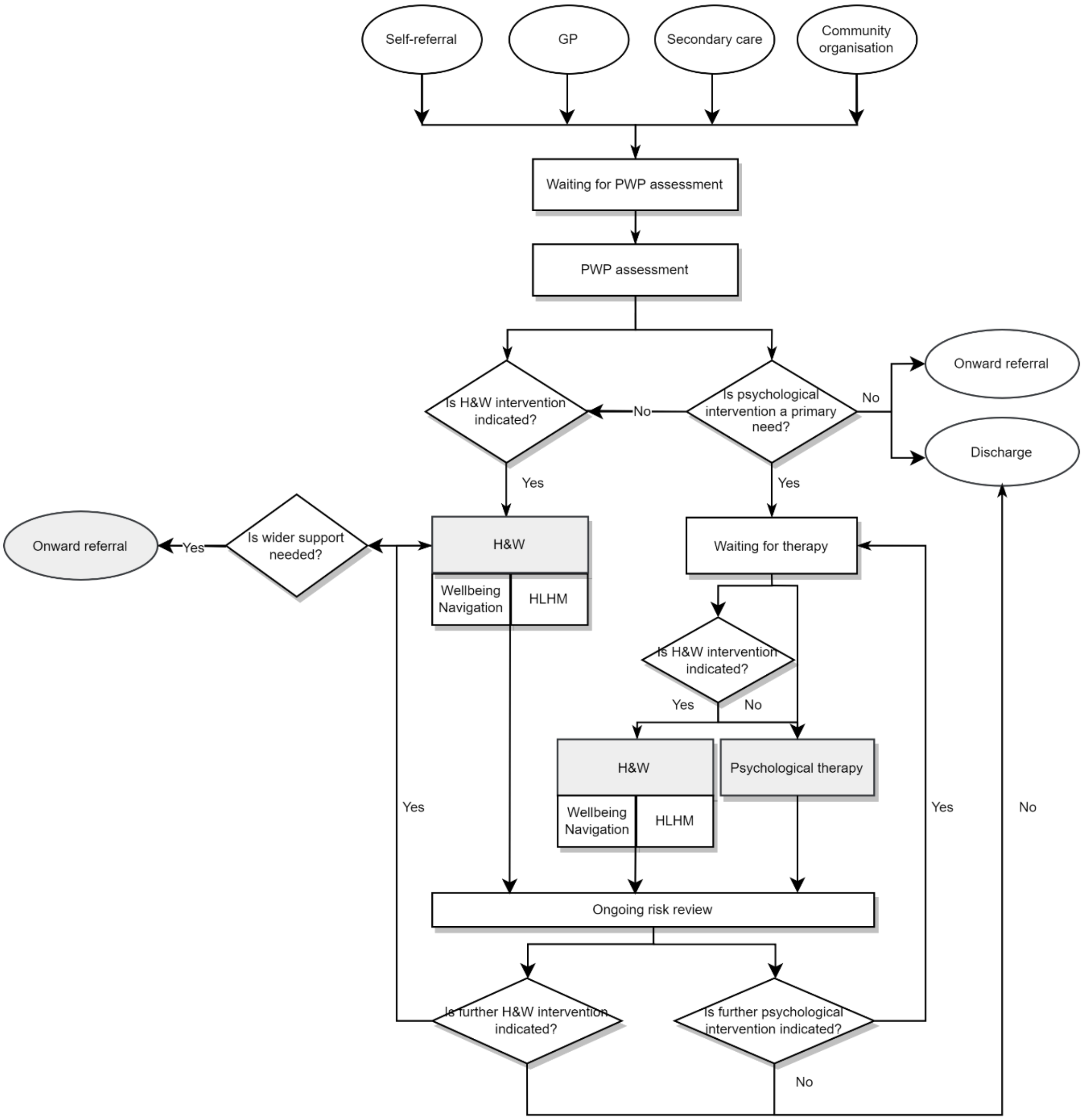


Grey shapes indicate the four types of support offered after PWP assessment; rectangle boxes indicate activities within IAPT; circles indicate activities outside of IAPT; diamonds indicate ongoing review questions.
Abbreviations: GP; General Practitioner, PWP; Psychological Wellbeing Practitioner, H&W; Health and Wellbeing Pathway, HLHM; Healthy Living Healthy Minds programme.

**Supplementary Table 1. The RECORD statement for pharmacoepidemiology (RECORD-PE) checklist of items, extended from the STROBE and RECORD statements, which should be reported in non-interventional pharmacoepidemiologic studies using routinely collected health data**

| Item No | STROBE items | RECORD items | RECORD-PE items | Page No |
|---|---|---|---|---|
| **Title and abstract** | | | | |
| 1 | (a) Indicate the study's design with a commonly used term in the title or the abstract.<br>(b) Provide in the abstract an informative and balanced summary of what was done and what was found. | 1.1: The type of data used should be specified in the title or abstract. When possible, the name of the databases used should be included.<br>1.2: If applicable, the geographical region and timeframe within which the study took place should be reported in the title or abstract.<br>1.3: If linkage between databases was conducted for the study, this should be clearly stated in the title or abstract. | — | 1-2 |
| **Introduction** | | | | |
| Background rationale | | | | |
| 2 | Explain the scientific background and rationale for the investigation being reported. | — | — | 4-5 |
| Objectives | | | | |
| 3 | State specific objectives, including any prespecified hypotheses. | — | — | 5 |
| **Methods** | | | | |
| Study design | | | | |
| 4 | Present key elements of study design early in the paper. | — | 4.a: Include details of the specific study design (and its features) and report the use of multiple designs if used.<br>4.b: The use of a diagram(s) is recommended to illustrate key aspects of the study design(s), including exposure, washout, lag and observation periods, and covariate definitions as relevant. | 5-6 Supplementary Figure 1 |
| Setting | | | | |
| 5 | Describe the setting, locations, and relevant dates, including periods of recruitment, exposure, follow-up, and data collection. | — | — | 5-8 |
| Participants | | | | |

For peer review only

| | | | | |
|---|---|---|---|---|
| 6 | (a) Cohort study—give the eligibility criteria, and the sources and methods of selection of participants. Describe methods of follow-up. Case-control study—give the eligibility criteria, and the sources and methods of case ascertainment and control selection. Give the rationale for the choice of cases and controls. Cross sectional study—give the eligibility criteria, and the sources and methods of selection of participants.<br>(b) Cohort study—for matched studies, give matching criteria and number of exposed and unexposed. Case-control study—for matched studies, give matching criteria and the number of controls per case. | 6.1: The methods of study population selection (such as codes or algorithms used to identify participants) should be listed in detail. If this is not possible, an explanation should be provided.<br>6.2: Any validation studies of the codes or algorithms used to select the population should be referenced. If validation was conducted for this study and not published elsewhere, detailed methods and results should be provided.<br>6.3: If the study involved linkage of databases, consider use of a flow diagram or other graphical display to demonstrate the data linkage process, including the number of individuals with linked data at each stage. | 6.1.a: Describe the study entry criteria and the order in which these criteria were applied to identify the study population. Specify whether only users with a specific indication were included and whether patients were allowed to enter the study population once or if multiple entries were permitted. See explanatory document for guidance related to matched designs. | 8 |
| Variables | | | | |
| 7 | Clearly define all outcomes, exposures, predictors, potential confounders, and effect modifiers. Give diagnostic criteria, if applicable. | 7.1: A complete list of codes and algorithms used to classify exposures, outcomes, confounders, and effect modifiers should be provided. If these cannot be reported, an explanation should be provided. | 7.1.a: Describe how the drug exposure definition was developed.<br>7.1.b: Specify the data sources from which drug exposure information for individuals was obtained.<br>7.1.c: Describe the time window(s) during which an individual is considered exposed to the drug(s). The rationale for selecting a particular time window should be provided. The extent of potential left truncation or left censoring should be specified.<br>7.1.d: Justify how events are attributed to current, prior, ever, or cumulative drug exposure.<br>7.1.e: When examining drug dose and risk attribution, describe how current, historical or time on therapy are considered.<br>7.1.f: Use of any comparator groups should be outlined and justified.<br>7.1.g: Outline the approach used to handle individuals with more than one relevant drug exposure during the study period. | 8-9 |
| Data sources/measurement | | | | |
| 8 | For each variable of interest, give sources of data and details of methods of assessment (measurement). Describe comparability of assessment methods if there is more than one group. | — | 8.a: Describe the healthcare system and mechanisms for generating the drug exposure records. Specify the care setting in which the drug(s) of interest was prescribed. | 8 |
| Bias | | | | |

For peer review only

| | | | | |
|---|---|---|---|---|
| 9 | Describe any efforts to address potential sources of bias. | — | — | 8<br>Table 1<br>Supplementary Figure 2 |
| Study size | | | | |
| 10 | Explain how the study size was arrived at. | — | — | 8-9 |
| Quantitative variables | | | | |
| 11 | Explain how quantitative variables were handled in the analyses. If applicable, describe which groupings were chosen, and why. | — | — | 9 |
| Statistical methods | | | | |
| 12 | (a) Describe all statistical methods, including those used to control for confounding.<br>(b) Describe any methods used to examine subgroups and interactions.<br>(c) Explain how missing data were addressed.<br>(d) Cohort study—if applicable, explain how loss to follow-up was addressed. Case-control study—if applicable, explain how matching of cases and controls was addressed. Cross sectional study—if applicable, describe analytical methods taking account of sampling strategy.<br>(e) Describe any sensitivity analyses. | — | 12.1.a: Describe the methods used to evaluate whether the assumptions have been met.<br>12.1.b: Describe and justify the use of multiple designs, design features, or analytical approaches. | 9-10 |
| Data access and cleaning methods | | | | |
| 12 | — | 12.1: Authors should describe the extent to which the investigators had access to the database population used to create the study population.<br>12.2: Authors should provide information on the data cleaning methods used in the study. | — | 9 |
| Linkage | | | | |
| 12 | — | 12.3: State whether the study included person level, institutional level, or other data linkage across two or more databases. The methods of linkage and methods of linkage quality evaluation should be provided. | — | n/a |
| **Results** | | | | |
| Participants | | | | |
| 13 | (a) Report the numbers of individuals at each stage of the study (eg, numbers potentially eligible, examined for eligibility, confirmed eligible, included in the study, completing follow-up, and analysed). | 13.1: Describe in detail the selection of the individuals included in the study (that is, study population selection) including filtering based on data quality, data availability, and | — | 9<br>Supplementary Table 2 |

For peer review only

| | | | | |
|---|---|---|---|---|
| | (b) Give reasons for non-participation at each stage.<br>(c) Consider use of a flow diagram. | linkage. The selection of included individuals can be described in the text or by means of the study flow diagram. | | |
| Descriptive data | | | | |
| 14 | (a) Give characteristics of study participants (eg, demographic, clinical, social) and information on exposures and potential confounders.<br>(b) Indicate the number of participants with missing data for each variable of interest.<br>(c) Cohort study—summarise follow-up time (eg, average and total amount). | — | — | 10<br>Table 2 |
| Outcome data | | | | |
| 15 | Cohort study—report numbers of outcome events or summary measures over time. Case-control study—report numbers in each exposure category, or summary measures of exposure. Cross sectional study—report numbers of outcome events or summary measures. | — | — | Table 2 |
| Main results | | | | |
| 16 | (a) Give unadjusted estimates and, if applicable, confounder adjusted estimates and their precision (eg, 95% confidence intervals). Make clear which confounders were adjusted for and why they were included.<br>(b) Report category boundaries when continuous variables are categorised.<br>(c) If relevant, consider translating estimates of relative risk into absolute risk for a meaningful time period. | — | — | 12<br>Table 3 |
| Other analyses | | | | |
| 17 | Report other analyses done—eg, analyses of subgroups and interactions, and sensitivity analyses. | — | — | 14<br>Table 4<br>Supplementary Table 3 |
| **Discussion** | | | | |
| Key results | | | | |
| 18 | Summarise key results with reference to study objectives. | — | — | 16-17 |
| Limitations | | | | |
| 19 | Discuss limitations of the study, taking into account sources of potential bias or imprecision. Discuss both direction and magnitude of any potential bias. | 19.1: Discuss the implications of using data that were not created or collected to answer the specific research question(s). Include | 19.1.a: Describe the degree to which the chosen database(s) adequately captures the drug exposure(s) of interest. | 17-18 |

For peer review only

| | | | | |
|---|---|---|---|---|
| | | discussion of misclassification bias, unmeasured confounding, missing data, and changing eligibility over time, as they pertain to the study being reported. | | |
| Interpretation | | | | |
| 20 | Give a cautious overall interpretation of results considering objectives, limitations, multiplicity of analyses, results from similar studies, and other relevant evidence. | — | 20.a: Discuss the potential for confounding by indication, contraindication or disease severity or selection bias (healthy adherer/sick stopper) as alternative explanations for the study findings when relevant. **[A: Original text indicated this item was RECORD (ie, not RECORD-PE)?]** | 19 |
| Generalisability | | | | |
| 21 | Discuss the generalisability (external validity) of the study results. | — | — | 18 |
| **Other information** | | | | |
| Funding | | | | |
| 22 | Give the source of funding and the role of the funders for the present study and, if applicable, for the original study on which the present article is based. | — | — | 1 |
| Accessibility of protocol, raw data, and programming code | | | | |
| 22 | — | 22.1: Authors should provide information on how to access any supplemental information such as the study protocol, raw data, or programming code. | — | n/a |

Abbreviations: RECORD=reporting of studies conducted using observational routinely collected data; RECORD-PE=RECORD for pharmacoepidemiologic research; STROBE=strengthening the reporting of observational studies in epidemiology.

For peer review only

**Supplementary Figure 2. Example box plots exploring the balance of covariates for age and baseline GAD-7 after propensity score estimation**

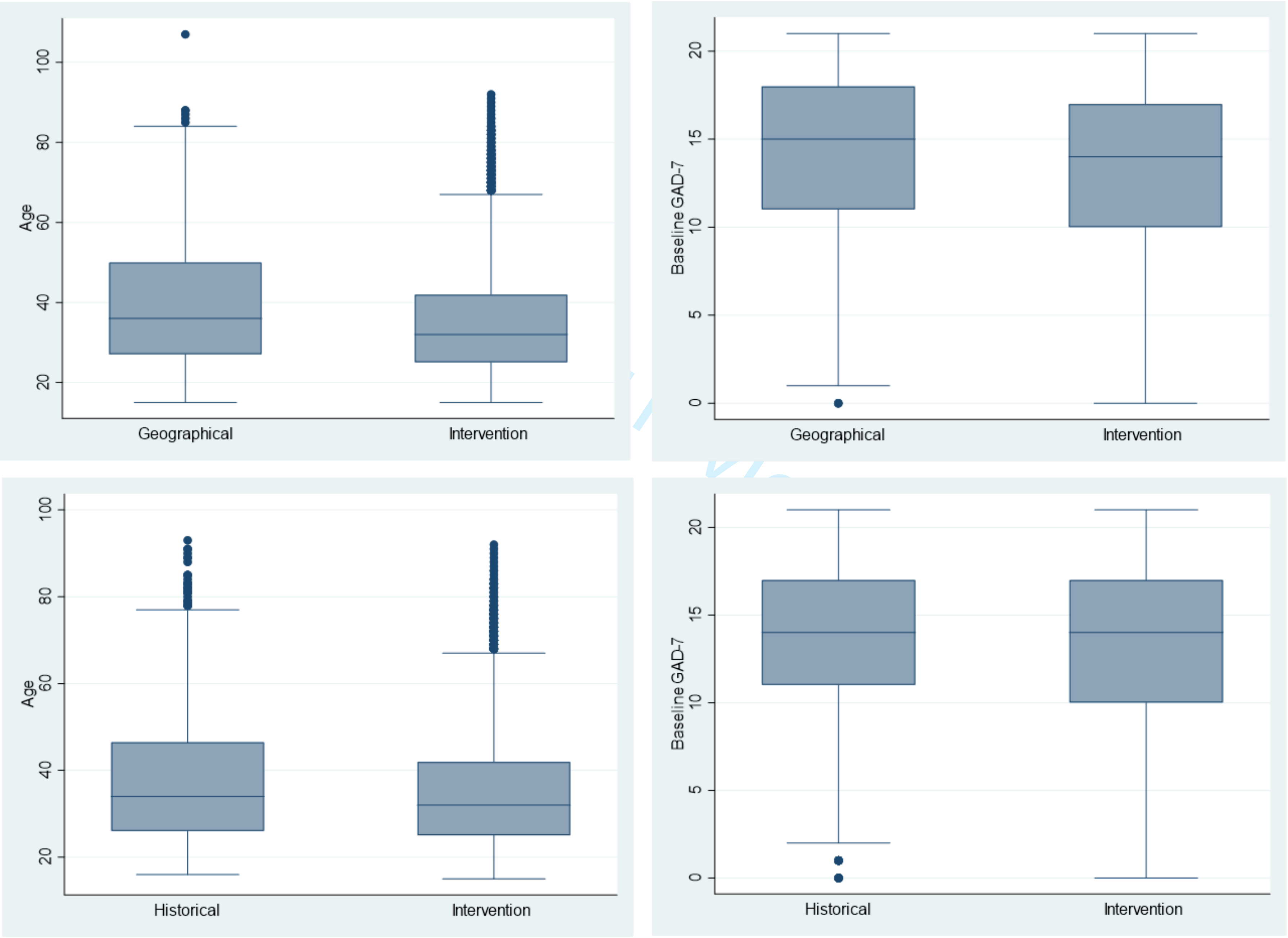

**Supplementary Table 2. Number of service-users included in analyses**

| | Intervention group | Geographical-control group | Historical-control group |
|---|---|---|---|
| In IAPT EHR dataset | 23,143 | 6865 | 12,045 |
| Eligible for analysis | 8020 | 3548 | 6074 |
| Eligible for analysis and have been discharged from service (as-started analysis) | 5853 | 2916 | 5372 |
| Eligible for analysis, have been discharged from service and have been within service for at least 12 weeks | 4284 | 1884 | 3698 |
| Eligible for analysis and have been discharged from service and have been within service for at least 16 weeks | 4529 | 1992 | 3738 |
| Eligible for analysis and have been discharged from service and have been within service for at least 20 weeks | 4153 | 1963 | 3630 |
| Eligible for analysis and have been discharged from service and have been within service for at least 24 weeks | 3747 | 1880 | 3287 |

Abbreviations: EHR; electronic health record

For peer review only

**Supplementary Table 3. Doubly robust mixed effects linear regression models exploring group by demographic interactions effects for primary outcome in as-started sample**

| | Intervention vs Geographical-control<br>n obs = 63,853, n patients = 11,271 | | | | Intervention vs Historical-control<br>n obs = 79,685, n patients = 13,748 | | | |
|---|---|---|---|---|---|---|---|---|
| | **Coefficient** | **95% CI** | | **p value** | **Coefficient** | **95% CI** | | **p value** |
| **PHQ-9** | | | | | | | | |
| Group X Sex | | | | | | | | |
| Intervention - Female | 0.39 | 0.07 | 0.71 | 0.017 | 0.15 | -0.11 | 0.42 | 0.256 |
| Group X Age category | | | | | | | | |
| Intervention - 29-40 years | 0.09 | -0.27 | 0.45 | 0.612 | 0.15 | -0.14 | 0.45 | 0.310 |
| Intervention - 41-91 years | 0.00 | -0.36 | 0.36 | 0.993 | 0.18 | -0.13 | 0.49 | 0.261 |
| Group X Ethnicity | | | | | | | | |
| Intervention - Non-White | 0.04 | -0.48 | 0.56 | 0.881 | -0.03 | -0.45 | 0.39 | 0.881 |
| Group X IMD category | | | | | | | | |
| Intervention - Intermediate | 0.24 | -0.16 | 0.63 | 0.238 | -0.20 | -0.50 | 0.10 | 0.199 |
| Intervention - High | 0.14 | -0.20 | 0.48 | 0.411 | -0.24 | -0.55 | 0.07 | 0.122 |
| **GAD-7** | | | | | | | | |
| Group X Sex | | | | | | | | |
| Intervention - Female | 0.38 | 0.09 | 0.66 | 0.010 | 0.11 | -0.12 | 0.35 | 0.345 |
| Group X Age category | | | | | | | | |
| Intervention - 29-40 years | 0.09 | -0.24 | 0.42 | 0.595 | 0.08 | -0.18 | 0.35 | 0.533 |
| Intervention - 41-91 years | -0.01 | -0.33 | 0.31 | 0.953 | 0.04 | -0.23 | 0.32 | 0.763 |
| Group X Ethnicity | | | | | | | | |
| Intervention - Non-White | -0.04 | -0.49 | 0.42 | 0.878 | -0.13 | -0.51 | 0.25 | 0.513 |
| Group X IMD category | | | | | | | | |
| Intervention - Intermediate | 0.09 | -0.26 | 0.44 | 0.610 | -0.23 | -0.50 | 0.04 | 0.089 |
| Intervention - High | -0.08 | -0.38 | 0.23 | 0.619 | -0.30 | -0.57 | -0.02 | 0.034 |
| **WSAS** | | | | | | | | |
| Group X Sex | | | | | | | | |
| Intervention - Female | 0.39 | -0.10 | 0.87 | 0.118 | 0.10 | -0.30 | 0.49 | 0.634 |
| Group X Age category | | | | | | | | |
| Intervention - 29-40 years | -0.15 | -0.71 | 0.40 | 0.586 | -0.27 | -0.72 | 0.17 | 0.222 |

For peer review only

| | | | | | | | | |
|---|---|---|---|---|---|---|---|---|
| Intervention - 41-91 years | -0.26 | -0.79 | 0.26 | 0.325 | 0.06 | -0.40 | 0.52 | 0.801 |
| Group X Ethnicity | | | | | | | | |
| Intervention - Non-White | -0.14 | -0.97 | 0.69 | 0.737 | 0.08 | -0.59 | 0.75 | 0.811 |
| Group X IMD category | | | | | | | | |
| Intervention - Intermediate | 0.19 | -0.40 | 0.78 | 0.533 | -0.19 | -0.64 | 0.26 | 0.414 |
| Intervention - High | -0.03 | -0.54 | 0.49 | 0.919 | -0.53 | -0.99 | -0.07 | 0.023 |

All models include main effects for group, age, sex, ethnicity and IMD and are adjusted for baseline PHQ-9, baseline GAD-7 and baseline WSAS. Inverse propensity score weighting applied to all models, weighted for all covariates. Each group by demographic interaction model was run separately but are presented together in this table.
Sample includes all patients who met the study inclusion criteria, with follow up as the point of service discharge.

For peer review only
